# High-density linkage map construction and QTL analysis for earliness-related traits in *Gossypium hirsutum* L

**DOI:** 10.1186/s12864-016-3269-y

**Published:** 2016-11-11

**Authors:** Xiaoyun Jia, Chaoyou Pang, Hengling Wei, Hantao Wang, Qifeng Ma, Jilong Yang, Shuaishuai Cheng, Junji Su, Shuli Fan, Meizhen Song, Nusireti Wusiman, Shuxun Yu

**Affiliations:** 1 College of Agronomy, Northwest A&F University, Yangling, 712100 China; 2 State Key Laboratory of Cotton Biology, Institute of Cotton Research of CAAS, Anyang, 455000 China; 3 Institute of Industrial Crops of Xinjiang Academy of Agricultural Sciences, Xinjiang, 830091 China

**Keywords:** *Gossypium hirsutum* L, Cotton earliness, Single nucleotide polymorphism (SNP), High-density genetic map, Quantitative trait loci (QTLs)

## Abstract

**Background:**

*Gossypium hirsutum* L., or upland cotton, is an important renewable resource for textile fiber. To enhance understanding of the genetic basis of cotton earliness, we constructed an intra-specific recombinant inbred line population (RIL) containing 137 lines, and performed linkage map construction and quantitative trait locus (QTL) mapping.

**Results:**

Using restriction-site associated DNA sequencing, a genetic map composed of 6,434 loci, including 6,295 single nucleotide polymorphisms and 139 simple sequence repeat loci, was developed from RIL population. This map spanned 4,071.98 cM, with an average distance of 0.63 cM between adjacent markers. A total of 247 QTLs for six earliness-related traits were detected in 6 consecutive years. In addition, 55 QTL coincidence regions representing more than 60 % of total QTLs were found on 22 chromosomes, which indicated that several earliness-related traits might be simultaneously improved. Fine-mapping of a 2-Mb region on chromosome D3 associated with five stable QTLs between Marker25958 and Marker25963 revealed that lines containing alleles derived from CCRI36 in this region exhibited smaller phenotypes and earlier maturity. One candidate gene (*EMF2*) was predicted and validated by quantitative real-time PCR in early-, medium- and late-maturing cultivars from 3- to 6-leaf stages, with highest expression level in early-maturing cultivar, CCRI74, lowest expression level in late-maturing cultivar, Bomian1.

**Conclusions:**

We developed an SNP-based genetic map, and this map is the first high-density genetic map for short-season cotton and has the potential to provide deeper insights into earliness. Cotton earliness-related QTLs and QTL coincidence regions will provide useful materials for QTL fine mapping, gene positional cloning and MAS. And the gene, *EMF2*, is promising for further study.

**Electronic supplementary material:**

The online version of this article (doi:10.1186/s12864-016-3269-y) contains supplementary material, which is available to authorized users.

## Background

*Gossypium hirsutum* L. (2*n* = 4*x* = 52), one of 50 *Gossypium* species and the leading natural fiber crop, contributes to more than 95 % of total cotton production [1, 2]. Short-season cotton, or early-maturity cotton, is a type of *G. hirsutum* with a relatively short growth period. Earliness, a distinctive characteristic of short-season cotton, is a complex agronomic trait of a quantitative genetic nature [3]. In terms of plant development, earliness may be described in reference to budding date, flowering timing (FT) and the whole growth period (WGP) [4]. Other agronomic traits, such as plant height (PH), node of the first fruiting branch (NFFB) and height of the NFFB (HNFFB), have also been used as earliness indexes [5, 6]. For example, Godoy and Palomo concluded that the lower the NFFB and the shorter the PH, the earlier the maturity [5]. To alleviate food crises, a large amount of farmland previously used for cotton planting is now devoted to food crops, especially in China. To exploit these limited natural resources via breeding programs and manage the competition between cereal crops and cotton through crop rotation, cotton earliness is attracting an increasing amount of attention from breeders [6].

Although a decrease in genetic diversity increases the difficulty of breeding by hybridization [7], marker-assisted selection (MAS) is a promising approach for cotton breeding because it shortens breeding cycles with more accuracy [8]. Following the publication of the first allotetraploid cotton genetic map in 1994 [1], a host of research programs have been dedicated to genetic map construction [9–15] and quantitative trait locus (QTL) mapping [16–19]. Because anonymous marker-based maps are becoming saturated, a genetic map based on conventional markers cannot achieve the resolution required for fine QTL mapping or positional cloning. Single nucleotide polymorphisms (SNPs) are highly abundant and suitable for high-density genetic mapping [20, 21]. Restriction-site associated DNA sequencing (RAD-seq) is a powerful and cost-effective method for detecting genetic variations [22, 23] and has become widely applied in plants [24–28]. Moreover, landmark breakthroughs in genome sequencing of cotton species such as *G. raimondii* [29, 30] *G. arboretum* [31], *G. barbadense* [32, 33] and *G. hirsutum* L. [34, 35], have been achieved and will facilitate the detection of SNP markers.

Compared with the number of studies evaluating fiber quality, limited investigations have been conducted to identify genetic signatures for earliness [36–43]. The genetic bases of earliness are poorly understood and present findings are insufficient for application to breeding practices. To provide additional information for breeding programs designed for specific traits, the molecular mechanisms underlying cotton earliness must thus be further investigated.

The research strengthes of our team in regard to short-season cotton were exploited to advance genetic understanding of earliness, thereby allowing us to construct immortalized recombinant inbred lines (RILs) consisting of 137 families from two *G. hirsutum* inbred lines: CCRI36 and G2005. The purpose of the present study was to (1) construct an intra-specific high-resolution genetic map for *G. hirsutum* using RAD-seq, (2) detect QTLs related to earliness for MAS in short-season cotton breeding and (3) lay a foundation for fine mapping and positional cloning for use in future studies.

## Results

### SNP detection and genetic map construction

For library construction, Taqα^I^ was chosen as the enzyme mainly because of its uniformly distributed cutting sites and sufficient coverage across the entire cotton genome (Additional file 1). We obtained 14,109,670 and 18,558,010 paired-end reads for CCRI36 and G2005, respectively, and 7,293,849 paired-end reads per line for the RIL population. The average Q30 score was 86.44 %, demonstrating the high quality of the sequences (Table 1). All read sequences generated in this study are available in the Sequence Read Archive (http://www.ncbi.nlm.nih.gov/Traces/sra/) under accession number PRJNA315785. For the genome sequence alignment of *G. hirsutum* [35], the depth of the parental materials reached 10.57× for G2005 and 6.43× for CCRI36. The average sequence depth of the 137 lines reached 4.59× (Table 1). We obtained 369,223 SNPs, of which 78,127 were polymorphic between the parents. The At sub-genome contained 227,397 SNPs, of which 43,869 (19.29 %) were polymorphic; the Dt sub-genome contained 141,826 SNPs, of which 34,258 (24.16 %) were polymorphic (Additional file 2). The percentage of marker polymorphisms on each chromosome varied from 3.4 % on chromosome A10 to 36.85 % on A6.

**Table 1:** Statistical description of sequence data obtained from the parents and the recombinant inbred line (RIL) population

Sample	Total Reads (M)	Average Q30(%)	Average GC content (%)	Average depth (×)	Coverage (%)
CCRI36	14.11	89.75	40.74	6.43	8.17
G2005	18.56	90.95	39.88	10.57	9.16
RILs	999.26	86.38	40.22	4.59	7.09

Q30 is a base call error rate of 0.001

**Fig. 1:**
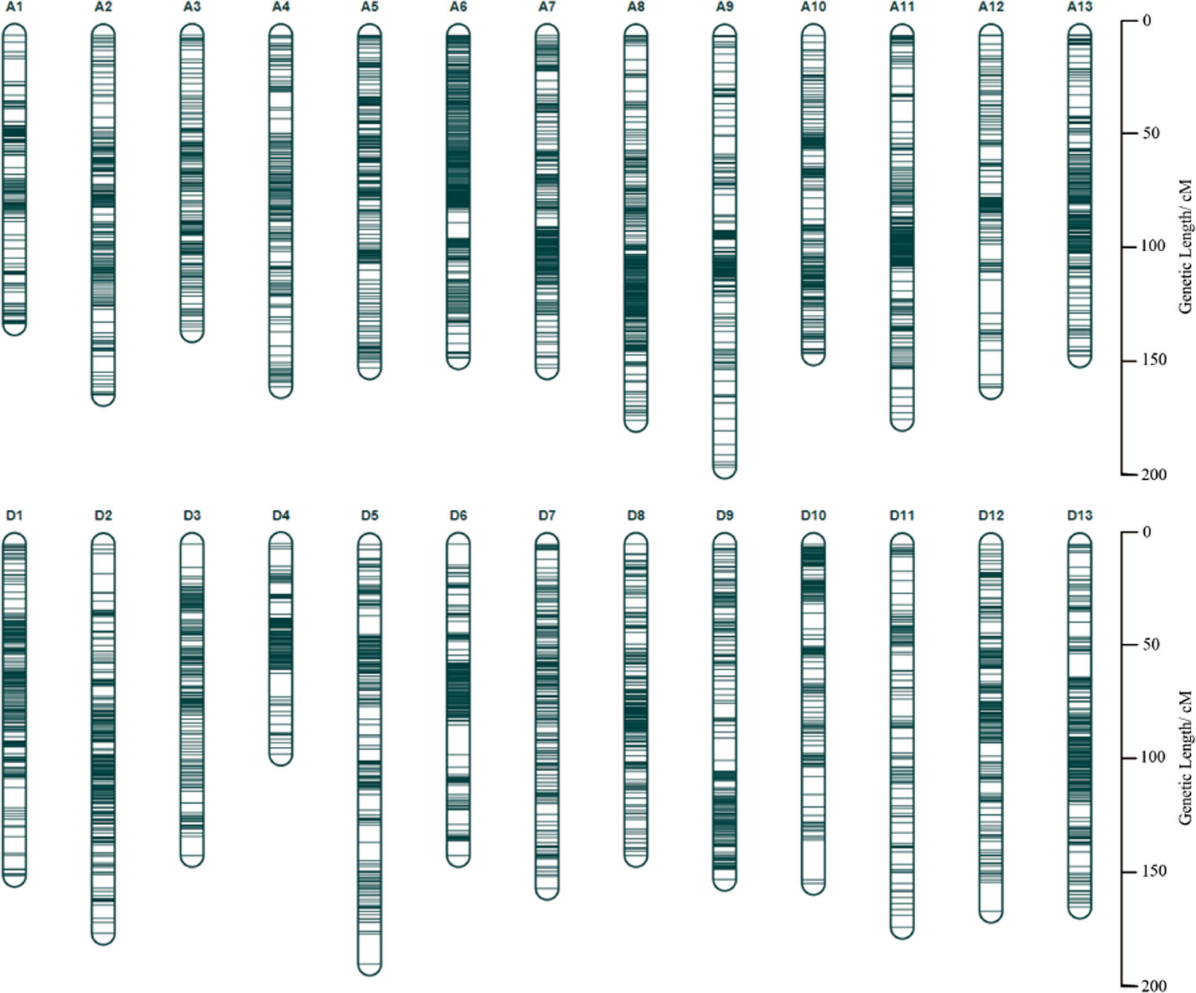
Linkage groups on the high-density genetic map. The13 chromosomes of the At sub-genome are shown on the upper part, the 13 chromosomes of the Dt sub-genome are shown on the lower part

For the construction of the genetic map, simple SNPs between the parents, represented as aa × bb, were selected in addition to the SSR markers. The final genetic map was composed of 6,434 loci including 6,295 simple SNPs and 139 SSR loci; it spanned 4,071.98 cM, with an average distance of 0.63 cM between adjacent markers (Fig. 1, Table 2, and Additional file 3). The At sub-genome contained a larger number of markers (3,536 SNPs and 64 SSR loci) compared with the Dt sub-genome (2,759 SNPs and 75 SSR loci). The average distance between adjacent markers was 0.57 cM in the At sub-genome and 0.71 cM in the Dt sub-genome. Marker distributions and linkage lengths varied among chromosomes. Chromosome A6 contained the most loci, 411, whereas D11 contained the fewest, 112. The largest linkage group was A9, with 196.04 cM; the shortest was D4, with 98.98 cM. The highest marker density was on A6, which had an average marker interval of only 0.36 cM. The largest gap among all chromosomes was 18.24 cM on D10.

**Table 2:** Detailed information on the single nucleotide polymorphism (SNP)-based genetic map

Chromosome	Loci	SNP loci	SSR loci	Length (cM)	Average interval (cM)	largest gap (cM)
A1	190	184	6	133.2	0.7	10.611
A2	218	216	2	163.92	0.76	7.254
A3	218	212	6	136.23	0.63	4.376
A4	169	169	0	160.85	0.95	7.19
A5	325	325	0	152.42	0.47	4.41
A6	411	399	12	147.89	0.36	7.192
A7	305	305	0	152.55	0.5	4.38
A8	386	386	0	175.59	0.45	6.65
A9	281	261	20	196.04	0.7	9.152
A10	258	258	0	146.32	0.57	4.89
A11	357	349	8	174.97	0.49	9.685
A12	157	154	3	160.88	1.03	15.37
A13	325	318	7	147.25	0.45	5.763
At sub-genome	3600	3536	64	2048.11	0.57	15.37
D1	257	240	17	152.01	0.59	9.246
D2	268	258	10	177.07	0.66	9.311
D3	179	179	0	143.18	0.8	6.65
D4	178	178	0	98.98	0.56	11.19
D5	252	241	11	190.57	0.76	13.703
D6	258	241	17	143.48	0.56	13.763
D7	208	208	0	157.77	0.76	5.48
D8	257	257	0	143.18	0.56	5.3
D9	201	189	12	153.85	0.77	10.321
D10	198	193	5	155.65	0.79	18.24
D11	112	109	3	174.54	1.57	8.071
D12	229	229	0	167.8	0.73	13.15
D13	237	237	0	165.79	0.7	10.72
Dt sub-genome	2834	2759	75	2023.87	0.71	18.24
Summary	6434	6295	139	4071.98	0.63	18.24

### Phenotypic variation

The mapping population was planted continuously from 2010 to 2015 at Anyang. We surveyed six earliness-related traits: WGP, FT, FBP, PH, NFFB and HNFFB.

Large differences existed between the parents, with G2005 exhibiting a later FT and NFFB appearance, a longer WGP and FBP, and a higher PH and HNFFB than CCRI36 (Additional file 4). All the six traits followed an approximately normal distribution and showed transgressive segregation in the RIL population, which indicated that the latter was a typical population for QTL detection (Additional file 4). Although been significantly affected by environmental effects, the broad-sense heritabilities of the six traits ranged from 74.93 % to 85.13 %, thereby demonstrating that the phenotypic variation was mainly genetically controlled and indicating in turn the potential for performing genetic modifications for earliness (Table 3). A simple correlation analysis showed that all six traits were positively correlated with one another (Additional file 5). FT was significantly positively correlated with the other five traits in all 6 years except with NFFB in 2011 and FBP in 2014 and 2015, in which significance levels of *p* = 0.05 or *p* = 0.01 were not reached. WGP was also significantly positively correlated with the other five traits in the 6 years except for with HNFFB in 2014. PH had a significant positive correlation with the other five traits except for with FBP in 2010 and 2014. FBP showed significant positive correlations with WGP and FT over the 6 years, with less significant correlations with PH, NFFB or HNFFB in 2010 and 2014. NFFB was significantly positively correlated with the other five traits over the years except for 2011; for this year, only the correlation between NFFB and HNFFB reached the *p* = 0.05 significance level. HNFFB was significantly positively correlated with the other five traits in the 6 years except for WGP and FBP in 2014. The results of the correlation analysis are consistent with the conclusion that the lower the NFFB and the shorter the PH, the earlier the maturity [5], and also suggest the high likelihood of gene pleiotropy or tightly linked functional genes.

**Table 3:** ANOVA analysis of the six earliness-related traits

Source of variation	FT	WGP	FBP	PH	HNFFB	NFFB
G	56.11***	293.87***	197.16***	314.98***	73.64***	5.76***
E	11081.84***	7202.56***	6488.76***	79418.15***	14480.68***	55.03***
G x E	9.43***	84.36***	68.92***	56.21**	14.18***	0.68***
error	3.37	20.93	18.76	44.27	8.98	0.27

*G* Genotype, *E* Environment, *FT* flowering timing, *WGP* whole growth period, *FBP* flowering to boll-opening period, *PH* plant height, *NFFB* node of the first fruiting branch, *HNFFB* height of the NFFB

** significant at the *p* < 0.01; *** significant at the *p* < 0.001

### QTL analysis

To reveal the genetic factors underlying cotton earliness, data for the six traits for the 6 years were analyzed separately and then combined using average values. A total of 247 QTLs were identified on all 26 chromosomes, including 39 QTLs for FT, 47 for WGP, 40 for FBP, 33 for PH, 43 for NFFB, and 45 for HNFFB (Additional file 6). Chromosome D3 contained 30 QTLs and ranked first among the 26 chromosomes, whereas A1, A6, D2, D6 and D11 contained fewer than 5 QTLs. A total of 52 stable QTLs were detected in at least two environments, and 52 QTLs accounted for more than 10 % of the phenotypic variation (PV). The paternal material G2005 and maternal material CCRI36 provided 146 and 101 of the trait-increasing alleles, respectively. Of the total QTLs, 109 were located on the At sub-genome and 138 were located on the Dt sub-genome. Nineteen stable QTLs were located on the At sub-genome, with the remaining 33 on the Dt sub-genome.

### WGP

WGP is important for successful cultivation and represents the first index for evaluating earliness because it directly indicates the extent of maturity [5]. Forty-seven QTLs for WGP were identified, including 10 stable and 6 major QTLs. Of the three stable QTLs located on D3, qGP-D3-4 was detected in 3 years and in the combined analysis. qWGP-D3-2 at 96.91 cM was detected in 2011 and 2012 and explained 12.33 %–18.27 % of the PV. qWGP-A12-1, detected in 2015 and in the combined analysis, respectively explained 15.24 % and 10.12 % of the observed PV.

### FT

Flowering is the transition from vegetative to reproductive stages and represents an efficient signal of earliness [44, 45]. For maximal yield, tailoring of FT in the crop life cycle is essential. In this study, a total of 39 QTLs were detected for FT, with 5 explaining 11.73 %-29.37 % of the PV and 6 being stably detected over time. qFT-D3-3 at 95.61–96.91 cM was stably detected in five years from 2011 to 2015 and in the combined analysis; it accounted for 19.14 %, 29.37 %, 21.31 % and 22.97 % of the PV in 2011, 2012, 2013 and the combined analysis, respectively. qFT-D3-2 at 86.81–87.11 cM was detected in 3 years and in the combined analysis and explained 25.99 % and 19.91 % of the PV in 2012 and the combined analysis, respectively. Application of FT as an indicator of cotton earliness revealed the fragment from 86.87–96.91 cM on D3 as an interesting candidate region, with CCRI36 conferred favorable alleles.

### FBP

FBP is a primary component of cotton development after flowering and directly affects harvesting rate. Forty QTLs were detected for FBP, with 10 stable QTLs detected in at least 2 environments and 9 explaining more than 10 % of the PV. The four QTLs, qFBP-A5-1, qFBP-D3-2, qFBP-D5-1 and qFBP-D7-3, were detected in three environments; the latter two QTLs explained more than 10 % of the PV. The QTL qFBP-D13-2 was detected in 2013 and 2015 and explained more than 10 % of the PV in both years.

### PH

PH is an important agronomic trait that influences plant architecture, production and mechanization. Thirty-three QTLs for PH were obtained, including 11 stable QTLs and 9 major QTLs that explained 10 %–21.77 % of the observed PV. Eight of the 11 stable QTLs were detected in the Dt sub-genome, and 6 were detected on chromosome D3. The five QTLs, qPH-D3-2 (at 57.11 cM), qPH-D3-3 (62.81–64.81 cM), qPH-D3-4 (83.81–87.11 cM), qPH-D3-5 (95.61–96.91 cM) and qPH-D12-3 (100.41–101.51 cM), were detected in three environments, with G2005 conferring the favorable allele increasing PH. qPH-D3-2 and qPH-D3-3, detected in 2013 and 2015 and in the combined analysis, explained 10.85 %–21.77 % and 7.51 %–14.96 % of the PV, respectively. qPH-D3-4 and qPH-D3-5 were detected in 2011, 2012 and 2014, and explained 6.03 %–11.27 % and 6.74 %–14.66 % of the PV, respectively. qPH-D12-3 was detected in 2013–2015, and explained 4.29 %–9.09 % of the PV.

### NFFB

NFFB has been suggested as an indicator of FT and a measure of relative photoperiodism [39]. A total of 43 QTLs were detected for NFFB in this study, with 12 major QTLs explaining 10.30 %–23.71 % of the PV. All nine stable QTLs were in the Dt sub-genome. qNFFB-D2-4 (at 134.11–134.91 cM) and qNFFB-D3-2 (96.91–101.91 cM) were detected in 3 years and in the combined analysis; they explained 6.59 %–13.10 % and 6.33 %–15.63 % of the PV, respectively. Another seven stable QTLs were detected in two environments.

### HNFFB

HNFFB, a primary component of PH, is significantly positively correlated with FT and WGP. Forty-five QTLs for HNFFB were detected, including 6 stable and 11 major QTLs. The four stable QTLs, qHNFFB-A4-1, qHNFFB-A7-1, qHNFFB-A11-3 and qHNFFB-A13-4, were located on the At sub-genome. Two notable QTLs, qHNFFB-D3-6 at 86.81–90.51 cM and qHNFFB-D3-7 at 96.91 cM, were detected in 2011, 2012 and 2015 and in the combined analysis; they explained 13.88 %–20.04 % and 17.03 %–24.71 % of the PV, respectively. G2005 conferred the favorable allele that increased HNFFB.

### QTL coincidence and gene annotation

The QTL mapping results indicated 55 QTL coincidence regions (with 95 % confidence intervals overlapped) on 22 chromosomes, and more than 60 % of all QTLs (158/247; 64.37 %) were involved in these regions. A total of 70.21 % of the WGP QTLs—the highest value among the six traits—were located in coincidence regions, followed by QTLs for HNFFB (68.89 %), FBP (67.50 %), PH (63.64 %), NFFB (62.79 %) and FT (51.28 %). Most QTLs in the same coincidence regions presented the same allelic direction, a phenomenon that explains the significant positive correlation among the six traits and is consistent with the speculation of gene pleiotropy or tightly linked functional genes (Table 4, Additional file 7). In addition, each trait had characteristic QTLs that might be useful for specific trait modification or ideotype breeding.

**Table 4:** Quantitative trait loci (QTL) for the six studied traits and QTLs located in coincidence regions

Trait	Total QTL	Coincided QTL	Percentage (%)
FT	39	20	51.28
FBP	40	27	67.50
WGP	47	33	70.21
NFFB	43	26	62.79
HNFFB	45	31	68.89
PH	33	21	63.64
total	247	159	64.37

*FT* flowering timing, *WGP* whole growth period, *FBP* flowering to boll-opening period, *PH* plant height, *NFFB* node of the first fruiting branch, *HNFFB* height of the NFFB

FT and FBP are two of the main components of WGP. FT and WGP had 10 common QTL coincidence regions that included 11 FT and 13 WGP QTLs, whereas FBP and WGP had 20 common coincidence regions that included 22 FBP and 22 WGP QTLs. In contrast, only six common coincidence regions were identified between FT and FBP; they included 7 FT and 7 FBP QTLs. HNFFB is a primary component of PH, and they shared 13 common QTL coincidence regions that included 17 HNFFB and 15 PH QTLs (Table 5). This phenomenon demonstrates the plasticity of earliness-related traits during developmental stages.

**Table 5:**
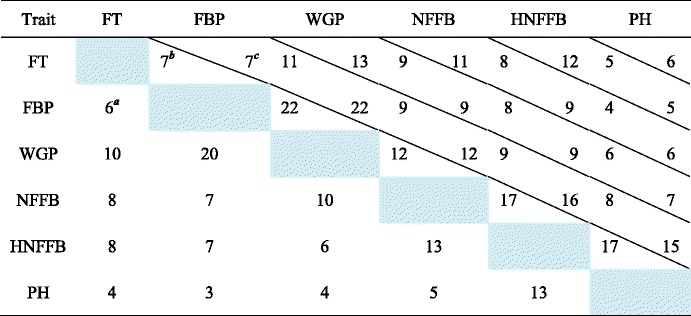
Number of coincidence regions and coincident quantitative trait loci (QTLs) between 15 trait pairs

*FT* flowering timing, *WGP* whole growth period, *FBP* flowering to boll-opening period, *PH* plant height, *NFFB* node of the first fruiting branch, *HNFFB* height of the NFFB

^***a***^ number of coincident QTLs between FT and FBP, ^***b***^ number of QTLs for FT in coincidence regions between FT and FBP, ^***c***^, number of QTLs number for FBP in coincidence regions between FT and FBP

**Fig. 2:**
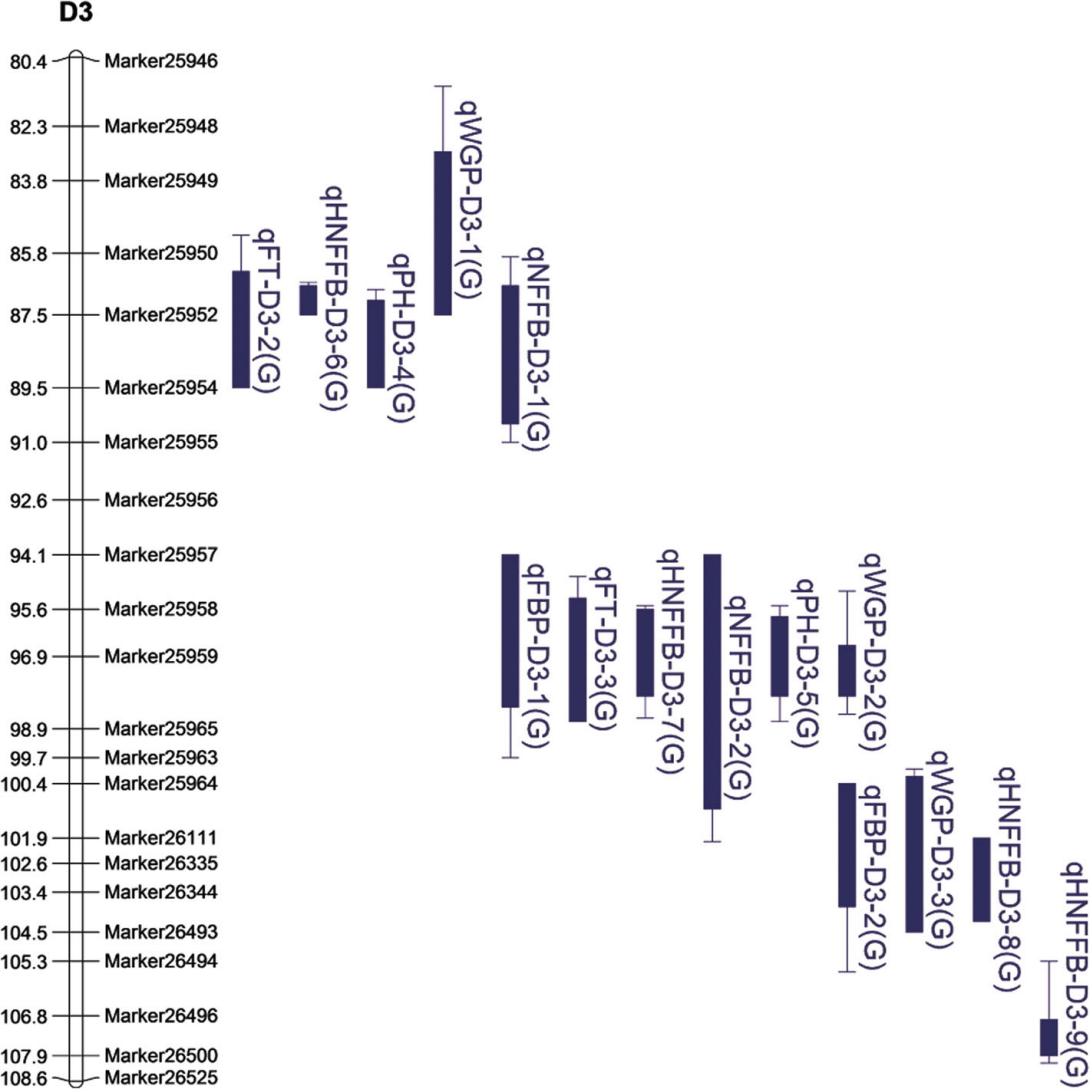
Quantitative trait loci detected on a noteworthy region of chromosome D3. FBP: flowering to boll-opening period; PH: plant height; FT: flowering timing; WGP: whole growth period; NFFB: node of the first fruiting branch; HNFFB: height of the NFFB

**Fig. 3:**
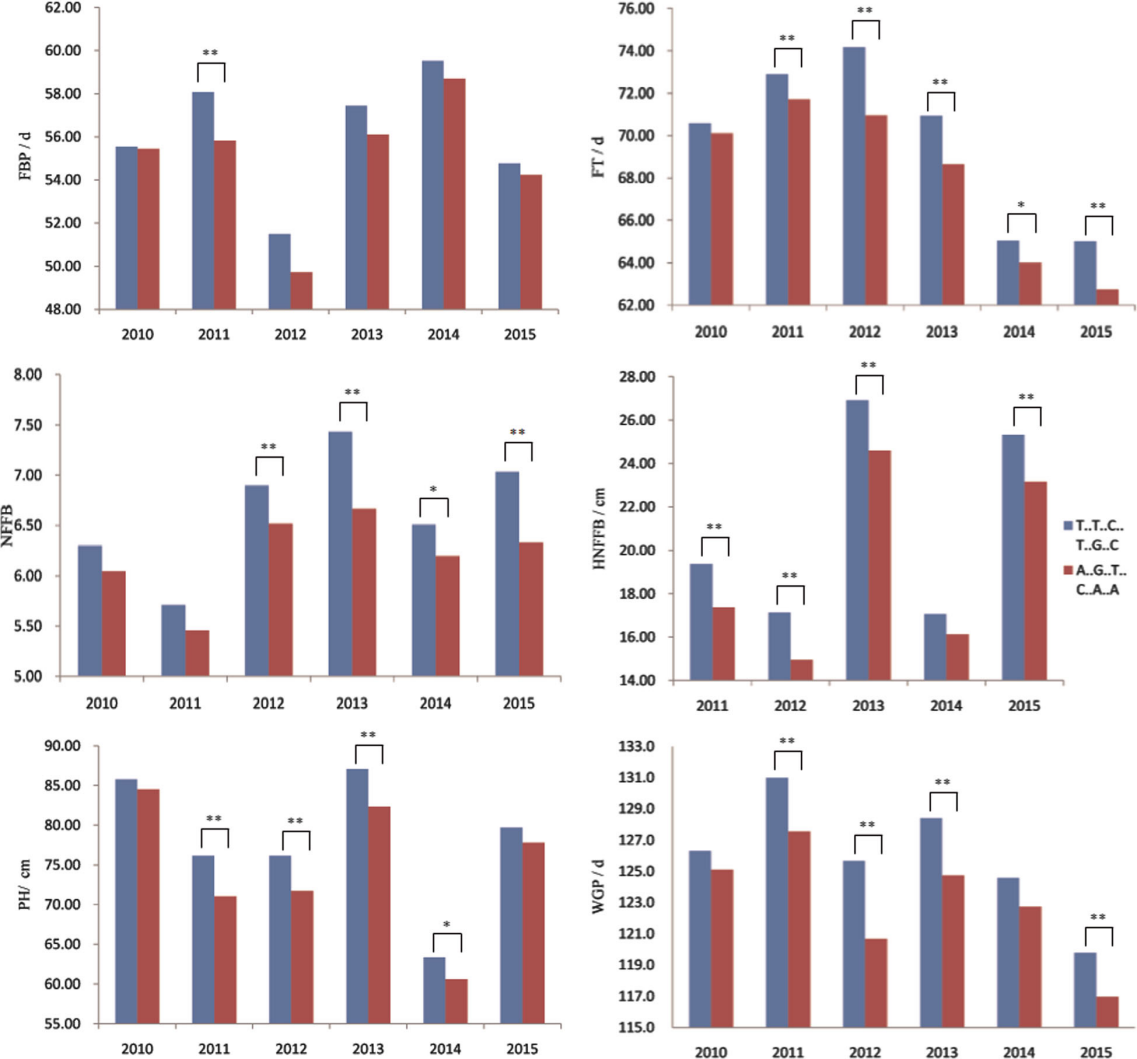
Phenotypic differences of six traits among six environments classified by the genotype of the locus at qFT-D3-3. The recombinant inbred line population was clearly divided into two phenotypes, and all six traits (node of the first fruiting branch [NFFB, A], plant height [PH, B], flowering to boll-opening period [FBP, C], height of the NFFB [HNFFB, D], flowering timing [FT, E], and whole growth period [WGP, F]) had the same trend in each environment. Blue and red bars respectively indicate the alleles of the six single nucleotide polymorphisms derived from G2005 (T..T..C..T..G..C) and CCRI36 (A..G..T..C..A..A). Data are shown as means. *, differences at *p* < 0.05; **, differences at *p* < 0.01

Among the 55 QTL coincidence regions, one chromosome region on D3 was extraordinary. Five stable QTLs were located in the core interval of 95.61–96.91 cM: qFT-D3-3, qWGP-D3-2, qPH-D3-5, qNFFB-D3-2 and qHNFFB-D3-7, which all had the same allele direction. In addition, qFBP-D3-1, which was detected in 2011, was also found in this region (Fig. 2, Additional file 8). Among the six QTLs, qFT-D3-3 was stably detected from 2011 to 2015 and was also detected in the combined analysis; thus, this QTL features high reliability for fine mapping. Six SNPs were mapped in the core region defined by Marker25957 and Marker25965. Based on the homozygous genotype of these six SNPs, we classified the 137 RILs into two groups. As expected, the lines inheriting the homozygous QTLs from CCRI36 flowered and matured earlier and had smaller phenotypes than those carrying the homozygous allele of G2005; in addition, all six traits exhibited stable phenotypic differences among the 6 years (Fig. 3). A total of 12 recombinants occurred in the RIL population at this locus, which allowed us to narrow down the interval to a 2-Mb region between Marker25958 and Marker25963 (Additional file 9). A total of 68 genes were annotated in this region (Additional file 10).

One gene, named *EMF2*, was primarily verified by qRT-PCR. The qRT-PCR analysis reflected that *EMF2* had the lowest expression level in the early-maturing cultivar CCRI74 and the highest in Bomian1, with Shan70 intermediate (Additional file 11). In all three cultivars, the expression level of *EMF2* decreased during the progression from the three- to the six true-leaf stage. This *EMF2* expression trend was consistent with that of flower buds differentiation in cotton. We therefore conclude that *EMF2* in cotton may also have a significant influence on flowering and is thus a potential candidate gene for use in further studies.

## Discussion

In this study, we selected CCRI36 and G2005 as parents for construction of a RIL population containing 137 lines. CCRI36 is an excellent early-maturity cultivar with high yield production. G2005 is a *G. barbadense* introgression line in the background of *G. hirsutum* and has a relatively longer growth period. This hybrid combination can both enrich the diversity of the intra-specific genetic mapping population and introduce desirable genes from *G. barbadense* into upland cotton for direct use on breeding, thus overcoming the difficulties of inter-specific crossing in cotton breeding [9]. The generated RIL population is therefore of great significance, both for theoretical genetic research and elite germplasm selection.

Genetic map is the first requisite for understanding the genetic mechanisms of a target trait. Rong et al. constructed a genetic map including 3,347 loci with 1.78 cM between adjacent markers [10]. Yu et al. published a genetic map with 2,316 loci and 1.91 cM in average marker distance [12]. Yu et al. reported a genetic map containing 2,072 loci with an average marker interval of 1.63 cM [13]. Liang et al. reported an updated map consisting of 3,414 loci with an average marker interval of 1.08 cM [14]. Shi et al. constructed a genetic map with 2,292 loci and 2.23 cM between adjacent markers [15]. Previously released genetic maps of *Gossypium* have primarily been based on PCR-based markers, which are laborious, time consuming and gradually less effective because of saturation during high-density genetic map construction. Since the application of high-throughput sequencing on cotton, marker density has been significantly increased. Wang et al. constructed an ultra-dense genetic map consisting of 4,999,048 SNPs distributed across 4,042 cM, and these authors used their high-density genetic map for genome assembly of *Gossypium hirsutum* acc. TM-1 [21]. Wang et al. applied the RAD-seq technique to upland cotton and identified 21,109 SNPs based on the genome sequence of *G. hirsutum* TM-1. They constructed a high-density genetic map comprising 3,984 RAD markers and 169 SSR markers and encompassing 3,500 cM, with 0.84 cM between adjacent markers [27]. Hulse-Kemp et al. constructed an ultra-high density genetic map using SNP arrays for marker detection and constructed a high-density genetic map consisting of 19,191 markers with 0.21 cM between adjacent markers [46]. Zhang et al. constructed a high-density genetic map that contained 5,521 SNPs with an average marker density of 0.78 cM [47]. Thus, high-throughput sequencing techniques are more efficient.

**Fig. 4:**
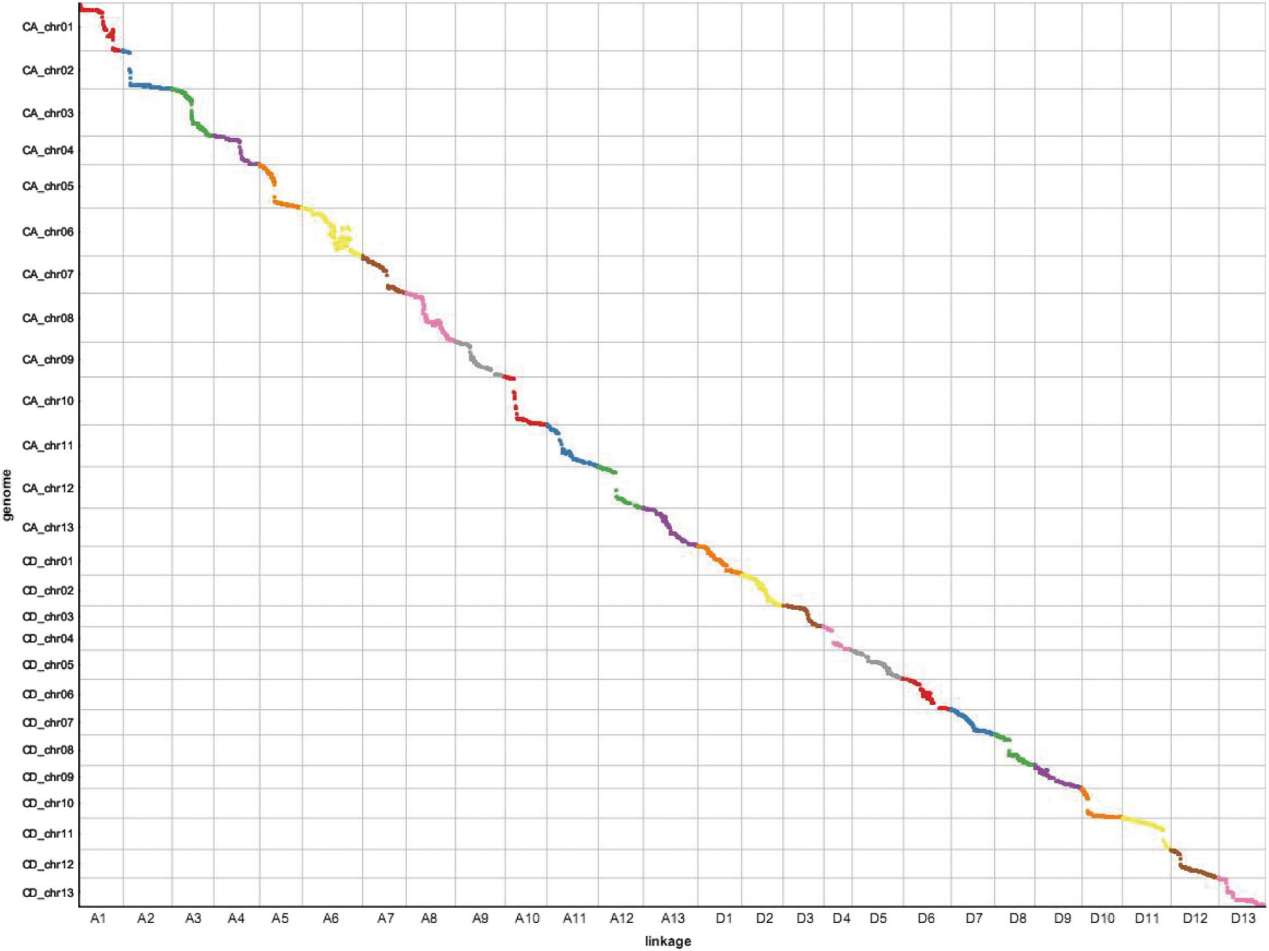
Marker collinearity between the genetic map and the reference genome. The x-axis indicates the arrangement of the 26 linkage groups (cM); the y-axis indicates the 26 reference chromosome sequences (Mb)

To construct a satisfactory high-density genetic map for short-season cotton, we adopted RAD-seq technology and developed 78,127 polymorphic SNPs between the parents. Polymorphisms between the parents reached 21.16 %, which was much higher than the value for SSR markers (Additional file 2). The genetic map, which contained 6,295 simple SNPs and 139 SSR loci, spanned 4,071.98 cM with an average of 0.63 cM between adjacent markers; this corresponds to a map length comparable to previous maps and a better marker density (Fig. 1, Table 2, Additional file 3). The mapped markers were uniformly distributed along the 26 cotton chromosomes (Additional file 12). The quality of our genetic map was evaluated by heat map analysis, which confirmed the accuracy of the 26 linkage groups because the recombination frequency among adjacent markers was relatively low (Additional file 13). In addition, linkage information for the 139 SSR loci was validated by a comparison with genetic maps reported by Liang et al. [14] and Blenda et al. [48]; in particular, the distribution of loci in their maps confirmed the common-marker-based assignment of the 26 linkage groups in our study (Additional file 14). Most of the marker collinearity between the genetic map and the genome sequence was clear, thus illustrating that the markers were accurately located on the present genetic map and had sufficient coverage on the 26 chromosomes (Fig. 4). Our map is the first high-density genetic map for short-season cotton based on SNP markers. In combination with the other published genetic maps described above, our map represents one of the most valuable resources available for cotton genome detection.

Published genetic studies on cotton have given priority to fiber quality [49], whereas few studies have focused on earliness. In this study, based on the high-density genetic map, we conducted QTL mapping for cotton earliness. The six studied traits were positively correlated with one another, which indicates the existence of gene pleiotropy or closely linked functional genes. This finding is consistent with the observation that more than half of detected QTLs (158/247; 64.37 %) were involved in QTL coincidence regions and that QTLs in the same coincidence region were characterized by the same allelic direction (Table 4, Additional file 4, Additional file 7); this phenomenon reveals the possibility of synchronously improving several earliness-related traits. FT and FBP are two of the main components of the WGP; however, the QTLs for these traits only partially coincided or overlapped. The same result was found between HNFFB and PH, with HNFFB is a primarily component of PH (Table 5). These limited common coincidence regions indicate that these genes might be expressed constitutively throughout the whole growth period or within the whole plant. In addition, various genes likely function at different developmental stages or in different parts of the plant, with each stage or organ modifiable by the ectopic expression of specific genes. Cotton earliness can therefore be specifically improved. And the needs of plants at different growth stages and environments must further be considered to support the most appropriate conditions for plant development.

Previous studies detected earliness-related QTLs distributed on almost all 26 cotton chromosomes [36–43]. In particular, QTLs for FBP, WGP, NFFB, HNFFB and PH were detected as follows: QTLs for FBP on chromosomes A3, A7, A8/D8, A9, A13, D3, D4 and D7 [38]; QTLs for WGP on chromosomes A13, D3 and D8 [38]; QTLs for NFFB on chromosomes A1, A5, A6, A9, A10, A11, A13, D1, D3, D6, D7, D9 and D11 [37, 39, 41]; QTLs for HNFFB on chromosomes A9, D3 and D6 [37]; and QTLs for PH on chromosomes A3, A4, A5, A9, A11, A13, D2, D3, D4, D5, D6, D8, D9 and D11 [36, 42]. These results indicate that D3 contains QTLs for FBP, WGP, NFFB, HNFFB and PH, which suggests that D3 is one of the chromosomes responsible for cotton earliness. Because of limitations in marker density and useful genome information, however, the published QTLs with large confidence intervals are not well understood and additional studies had not been reported prior to the present investigation. By analyzing the high-density genetic map, we identified more than 200 QTLs, including 30 QTLs on D3. A stable QTL at 95.61 cM, qFT-D3-3, was identified in six environments and coincided with another five QTLs in the interval of 95.61–96.91 cM. This result demonstrates that this region may contain a critical gene(s) tightly related to earliness (Fig. 2). Several other QTLs were also detected upstream or downstream of this region (Fig. 2, Additional file 8). Homozygous RILs in this interval were clearly classifiable into two groups with different phenotypes. The first group, which inherited homozygous QTL alleles from the G2005 cultivar, exhibited larger plant architectures and later flowering. The other group, which inherited alleles from the CCRI36 cultivar, was characterized by smaller phenotypes and earlier flowering (Fig. 3). On the basis of 12 recombinants in the RIL population, we narrowed down the core interval to a region spanning approximately 2 Mb between Marker25958 and Marker25963 (Additional file 9). Sixty-eight genes were annotated in this interval; one of them, *EMF2*, was regularly expressed among cultivars with different growth periods (Additional file 10). At all four stages, *EMF2* displayed relatively low expression in CCRI74 compared with Shan70, but showed the highest expression in Bomian1 (Additional file 11). Earlier studies found that *EMF2* is involved in various pathways, including delayed flowering [50–52]. We speculate that *EMF2* in cotton also has a major role in flowering. Because of limitations related to population type or size, however, further pinpointing the candidate interval based on the 137-RIL population will be difficult. New populations are consequently needed, such as near isogenic lines (NILs) and larger secondary F_2_ populations. And we plan to perform additional work involving on QTL fine mapping and functional verifications in future studies.

## Conclusions

In this study, we developed a high-density genetic map, the first SNP-based map constructed for short-season cotton that will be useful for genetic studies of earliness. More than 200 QTLs were detected and 55 QTL coincidence regions were analyzed; these results will provide useful materials for QTL fine mapping, gene positional cloning and MAS. One gene, *EMF2*, was verified by qRT-PCR among three cultivars from 3- to 6-leaf developmental stages; the result demonstrated this gene is promising for further study. In addition, the approach used in this study, RAD-seq is a powerful technique for SNP detection in high-resolution genetic map construction with polyploidy species.

## Methods

### Mapping population and statistical analysis

*Gossypium hirsutum* accession CCRI36 and an introgression line from *G. barbadense* G2005 were used as parental materials in a cross in 2006. CCRI36 and G2005 are upland cotton cultivars/lines bred by our lab. F_1_ seeds were planted in Hainan, China, in the winter of 2006. A total of 137 F_2_ plants were randomly selected and self-mated at Anyang, Henan province, China, in 2007. Beginning from the F_2:3_ generation, single-seed descent was conducted until the F_2:9_ generation to construct a RIL population. All RILs and parents were planted on the farm of the Cotton Research Institute of the Chinese Academy of Agricultural Sciences at Anyang from 2010 to 2015. A randomized complete block design was adopted with three replicates per year, with 25 plants retained in 5-m-long rows spaced 80 cm apart. We surveyed six earliness-related traits: PH (height from the ground to the main stem tip), NFFB (node number of the first fruiting branch, with the cotyledonary node recorded as zero), HNFFB (height from the ground to the NFFB), WGP (the number of days from the date of sowing to the date when 50 % of plants reached boll opening), FT (the number of days from the date of sowing to the date when 50 % of plants were flowering), and flowering to boll-opening period (FBP, the number of days from flowering to boll opening). All of the plants were used for the WGP, FT and FBP surveys, whereas 10 plants in the middle of each row were selected for the NFFB, HNFFB and PH measurements.

Phenotypic data were analyzed using SAS software (SAS Institute, Inc., Cary, NC, USA). The PROC GLM procedure in SAS was used to estimate the variance. An analysis of variance was performed for the yearly data, and the results over 6 years were combined. Broad-sense heritability was calculated according to Knapp et al. [53].

Three cultivars were used for gene validation: CCRI74, Shan70 and Bomian1. CCRI74 is an early-maturing cultivar with a 105-day WGP, Bomian1 is a late-maturing cultivar with a 135-day WGP, and Shan70 is an intermediate-maturing cultivar with a 117-day WGP.

### DNA extraction and simple sequence repeat (SSR) analysis

Genomic DNA was extracted from young leaves of the 137-RIL population and the two parents using the CTAB method [54]. A total of 3,624 SSR primer pairs were previously screened for polymorphisms between the parents; of these, 152 polymorphic primers were used to genotype the RIL population. All primer sequences used in this study can be downloaded from CottonGen (https://www.cottongen.org/). The G2005 genotype was designed as A, the CCRI36 genotype was designed as B and the heterozygous genotype was designed as H. If an SSR primer amplified more than one polymorphic band, a suffix of a/b/c was added after the primer name according to the product length from longest to shortest.

### Bar-coded library preparation

Bar-coded libraries were created as described by Andolfatto et al. [55] with several modifications, with 6-bp barcoded adapters designed and modified according to the standard Illumina adapter protocol for paired-end read libraries. DNA was enzyme digested using FastDigest Taqα^I^ (Thermo scientific Fermentas) and adapter ligation was carried out with T4 DNA ligase (Enzymatics). Subsequently, 19 to 24 of the resulting samples with different indexes were pooled together. DNA fragments between 400 and 600 bp were retrieved and purified using a QIAquick Gel Extraction kit (Qiagen, Valencia, CA). After purification, the adapter-ligated DNA fractions were subjected to PCR amplification (Phusion High-fidelity, Finnzymes). The PCR products were gel separated, and the 400–600-bp DNA fractions were purified using a QIAquick PCR Purification kit (Qiagen, Valencia, CA). Finally, the purified libraries were quantified on an Agilent 2100 Bioanalyzer and sequenced on an Illumina HiSeq 2000 instrument at BGI, Shenzhen, China.

### Sequence alignment and SNP identification

Genomic libraries were prepared and sequenced on the Illumina HiSeq 2000 platform according to the manufacturer’s instructions. Reads from multiple Illumina sequence channels were first filtered and segregated with the appropriate adapters assigned to each sample. Sequence alignment and SNP discovery were realized using the method described by Chen et al. [56]. All qualified sequences were mapped back to genome sequences of *G. hirsutum* [35] using the short-read sequences alignment software program BWA (version 0.5.9-r16) [57]. The Genome Analysis Toolkit (GATK) was used as the best practical approach for variation detection, with SNPs that were common between SAMTools and GATK considered to be candidates [58, 59]. Given the RIL population, SNPs that were homozygous in both parents were used to construct the genetic map. We selected single-dose SNPs that were only present on one homologous chromosome.

### Map construction and QTL analysis

Polymorphic SNPs were refiltered prior to map construction. SNPs were removed if they met any of the following five criteria: 1) the SNP was missing in more than 20 % of RILs; 2) the *p*-value of the segregation distortion was less than 0.01; 3) the sequencing depth of the parents was less than 4×; 4) either of the parents was heterozygous (heterozygosity > 20 %); and 5) the SNPs were redundant and in the same reads. Map construction was performed with a HighMap strategy as detailed in Liu [60]. The input datasets included the SSR marker and SNP genotypes. First, the high-quality markers were clustered into linkage groups based on a pair-wise modified logarithm of the odds (LOD) score for the recombination frequency. Second, Gibbs sampling, spatial sampling, and a simulated annealing algorithm were used to order the markers and estimate map distances. Third, genotyping errors (singletons) were identified and eliminated according to parental contributions to the genotypes using the k-nearest neighbor algorithm. To order the markers correctly, ordering and error correction processes were conducted iteratively. After several cycles, high-density linkage maps were obtained. Finally, heat maps were constructed and SNP collinearity was assessed to evaluate the map quality. The Kosambi function was used to convert the recombination frequencies into centimorgans.

For the QTL analysis, phenotypic data from 6 years were used separately and in combination. The combined analysis was conducted using the average of the phenotypic data. The QTLs were analyzed by composite interval mapping using WinQTLCart 2.5. The parameters were set to 5 cM of the window size and 1 cM of the walk speed, with up to 10 background markers. A LOD score ≥ 2.5 was used to detect QTLs. QTLs at the same location for the same trait across different years or in the same environments were regarded as ‘stable’, with QTLs explaining more than 10 % of the phenotypic variation (PV) regarded as ‘major’. QTLs were named according to McCouch et al. [61].

### Gene annotation and quantitative real-time PCR (qRT-PCR)

Genes in the target region were annotated using the annotation information reported by Tianzhen Zhang [35]. Total RNA was extracted from the apical buds of CCRI74, Shan70 and Bomian1 cultivars at three- to six-leaf stages using an RNAprep Pure Kit for Plants (Tiangen, China). First-strand cDNA synthesis was performed on 2 μg of total RNA using a First Strand cDNA Synthesis kit (Toyobo, Japan). The synthesized cDNA samples were diluted 10-fold and then used as cDNA templates for the qRT-PCR analysis. The qRT-PCR analysis was conducted using an Applied Biosystems 7500 Fast Real-Time PCR System and Fast SYBR Green Master Mix (Life Technologies, Foster City, CA, USA) with three technical replicates.

## Additional files

Additional file 1: Distribution of the TaqαI enzyme recognition site on the 26 cotton chromosomes. (TIF 1211 kb)
Additional file 2: Genotypes of the 6,295 polymorphic SNPs between the parents and SNP polymorphisms on each chromosome. (XLSX 221 kb)
Additional file 3: Detailed marker positions and linkage length of the 26 linkage groups. (XLSX 143 kb)
Additional file 4: Phenotypic distribution for the six traits of the parents and the RIL population. FBP: flowering to boll-opening period; PH: plant height; FT: flowering timing; WGP: whole growth period; NFFB: node of the first fruiting branch; HNFFB: height of the NFFB. (XLS 30 kb)
Additional file 5: Correlation coefficient (r) matrices for the six traits measured in the RIL population. FBP: flowering to boll-opening period; PH: plant height; FT: flowering timing; WGP: whole growth period; NFFB: node of the first fruiting branch; HNFFB: height of the NFFB. (XLS 29 kb)
Additional file 6: Detailed information of the 247 QTLs for the six cotton earliness-related traits from the RIL population. Stable or major QTLs are marked in yellow. (XLS 84 kb)
Additional file 7: QTL and QTL coincidence region distributions on the genetic map. QTLs located in the coincidence regions are marked in blue. (PDF 343 kb)
Additional file 8: The LOD score of QTLs coinciding with qFT-D3-3 on chromosome D3. FBP: flowering to boll-opening period; PH: plant height; FT: flowering timing; WGP: whole growth period; NFFB: node of the first fruiting branch; HNFFB: height of the NFFB. (TIF 3549 kb)
Additional file 9: Recombinants in the RIL population at locus qFT-D3-3 defining the core region between Marker25957 and Marker25965. CCRI36 is the maternal cultivar; G2005 is the paternal line; b is the homozygous allele conferred by CCRI36; a is the homozygous allele conferred by G2005; and h is heterozygous. (PDF 124 kb)
Additional file 10: Genes annotated between Marker25958 and Marker25963. (XLS 65 kb)
Additional file 11: EMF2 expression levels in apical buds of CCRI74, Shan70 and Bomian1. RNA was extracted during three- to six-true-leaf developmental stages. CCRI74, Shan70 and Bomian1 are respectively early-, intermediate- and late-maturing cultivars. Data are represented as means. (TIF 82 kb)
Additional file 12: Distribution of the mapped SNPs on the 26 reference chromosomes. The horizontal line indicates the physical position (Mb) of the 26 chromosomes, and the vertical line indicates the 26 chromosomes from D13 to A1. (TIF 1351 kb)
Additional file 13: Heat maps of the 26 linkage groups. (PDF 4231 kb)
Additional file 14: SSR locus positions on the genetic map and common markers between our map and the references. (XLS 40 kb)

## Abbreviations

FBP: Flowering to boll-opening period
FT: Flowering timing
HNFFB: Height of NFFB
NFFB: Node of first fruiting branch
PH: Plant height
PV: Phenotypic variation
qRT-PCR: Quantitative real-time PCR
QTL: Quantitative trait locus
RAD-seq: Restriction-site associated DNA sequencing
RIL: Recombinant inbred line
SNP: Single nucleotide polymorphism
WGP: Whole growth period.
