# Supplementary figures and images for: High-density linkage map construction and QTL analysis for earliness-related traits in Gossypium hirsutum L
Source: BMC Genomics. 2016 Nov 11;17:909. doi: 10.1186/s12864-016-3269-y (PMC5106845; doi:10.1186/s12864-016-3269-y)

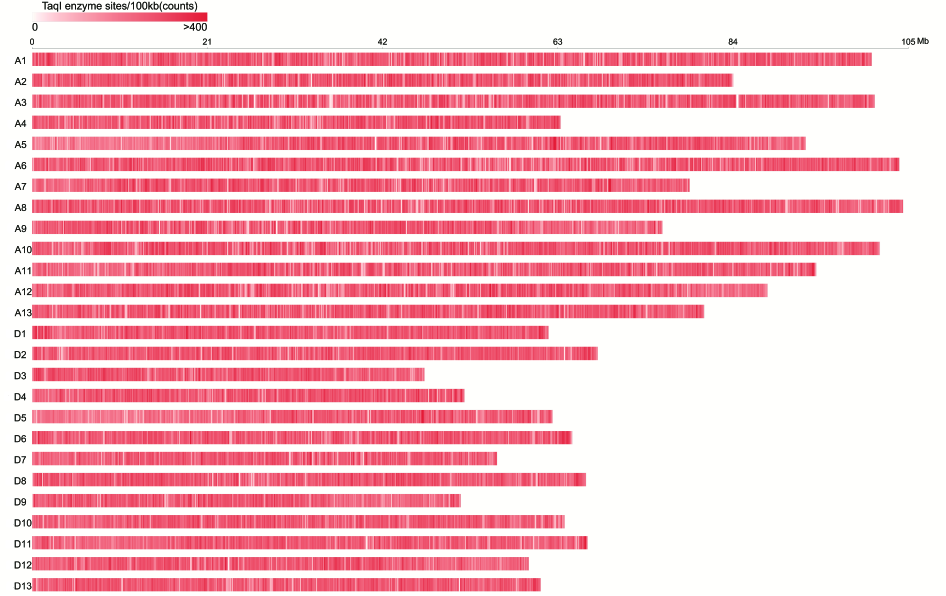

Supplement: Additional file 1: — Distribution of the TaqαI enzyme recognition site on the 26 cotton chromosomes. (TIF 1211 kb) [file 12864_2016_3269_MOESM1_ESM.tif]

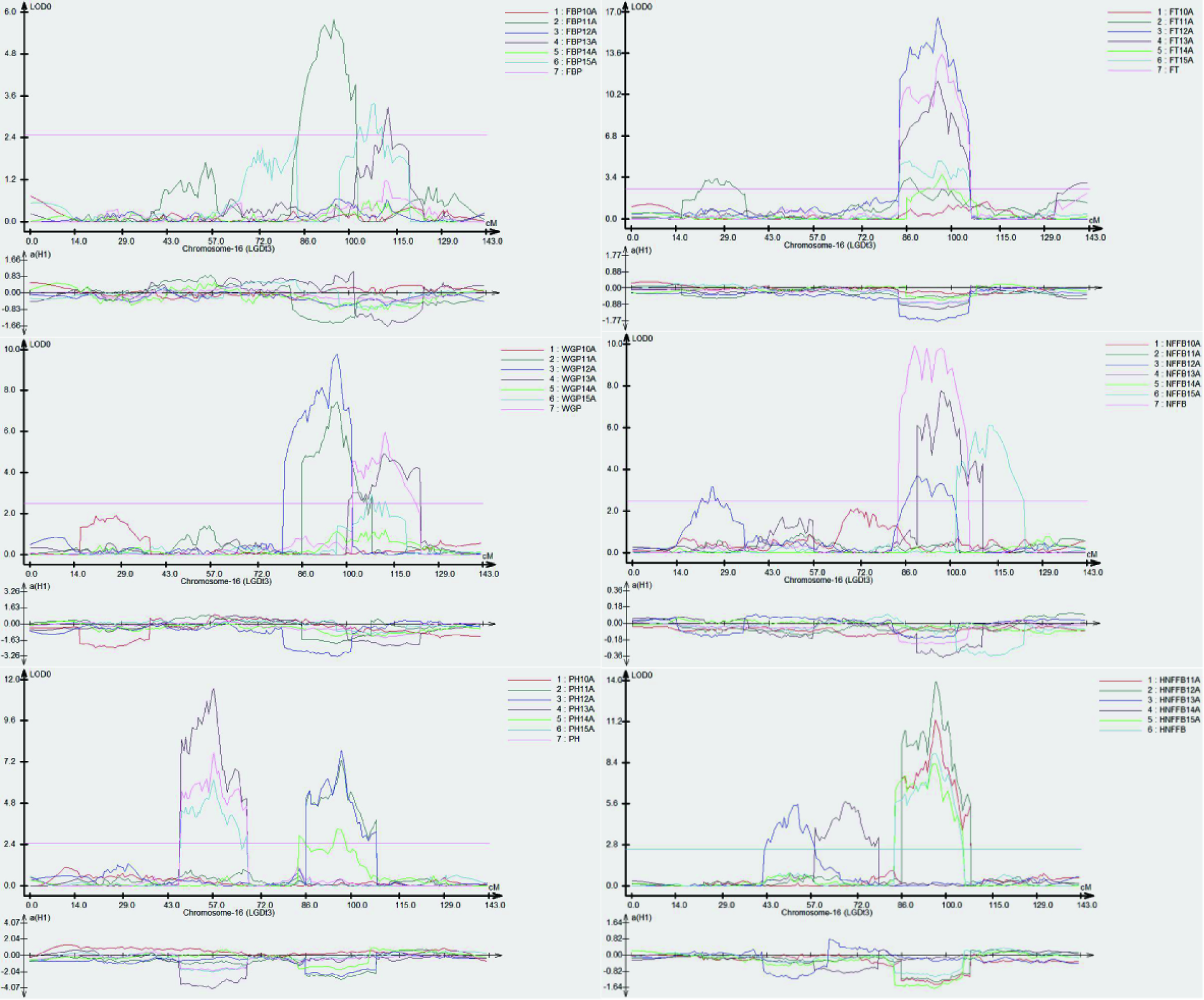

Supplement: Additional file 8: — The LOD score of QTLs coinciding with qFT-D3-3 on chromosome D3. FBP: flowering to boll-opening period; PH: plant height; FT: flowering timing; WGP: whole growth period; NFFB: node of the first fruiting branch; HNFFB: height of the NFFB. (TIF 3549 kb) [file 12864_2016_3269_MOESM8_ESM.tif]

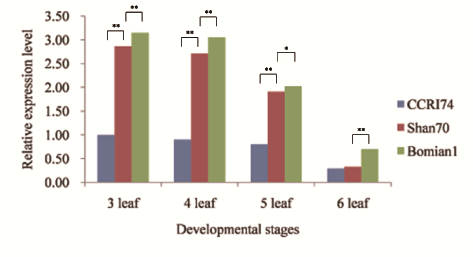

Supplement: Additional file 11: — EMF2 expression levels in apical buds of CCRI74, Shan70 and Bomian1. RNA was extracted during three- to six-true-leaf developmental stages. CCRI74, Shan70 and Bomian1 are respectively early-, intermediate- and late-maturing cultivars. Data are represented as means. (TIF 82 kb) [file 12864_2016_3269_MOESM11_ESM.tif]

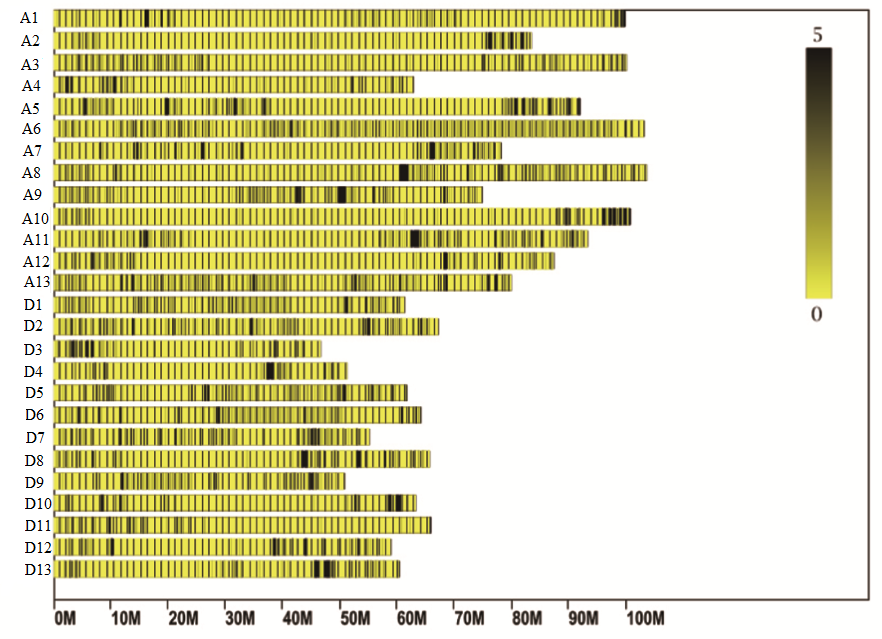

Supplement: Additional file 12: — Distribution of the mapped SNPs on the 26 reference chromosomes. The horizontal line indicates the physical position (Mb) of the 26 chromosomes, and the vertical line indicates the 26 chromosomes from D13 to A1. (TIF 1351 kb) [file 12864_2016_3269_MOESM12_ESM.tif]
