# Supplementary material for: High-density linkage map construction and QTL analysis for earliness-related traits in Gossypium hirsutum L
Source: BMC Genomics. 2016 Nov 11;17:909. doi: 10.1186/s12864-016-3269-y (PMC5106845; doi:10.1186/s12864-016-3269-y)
Supplement: Additional file 7: — QTL and QTL coincidence region distributions on the genetic map. QTLs located in the coincidence regions are marked in blue. (PDF 343 kb) [file 12864_2016_3269_MOESM7_ESM.pdf]

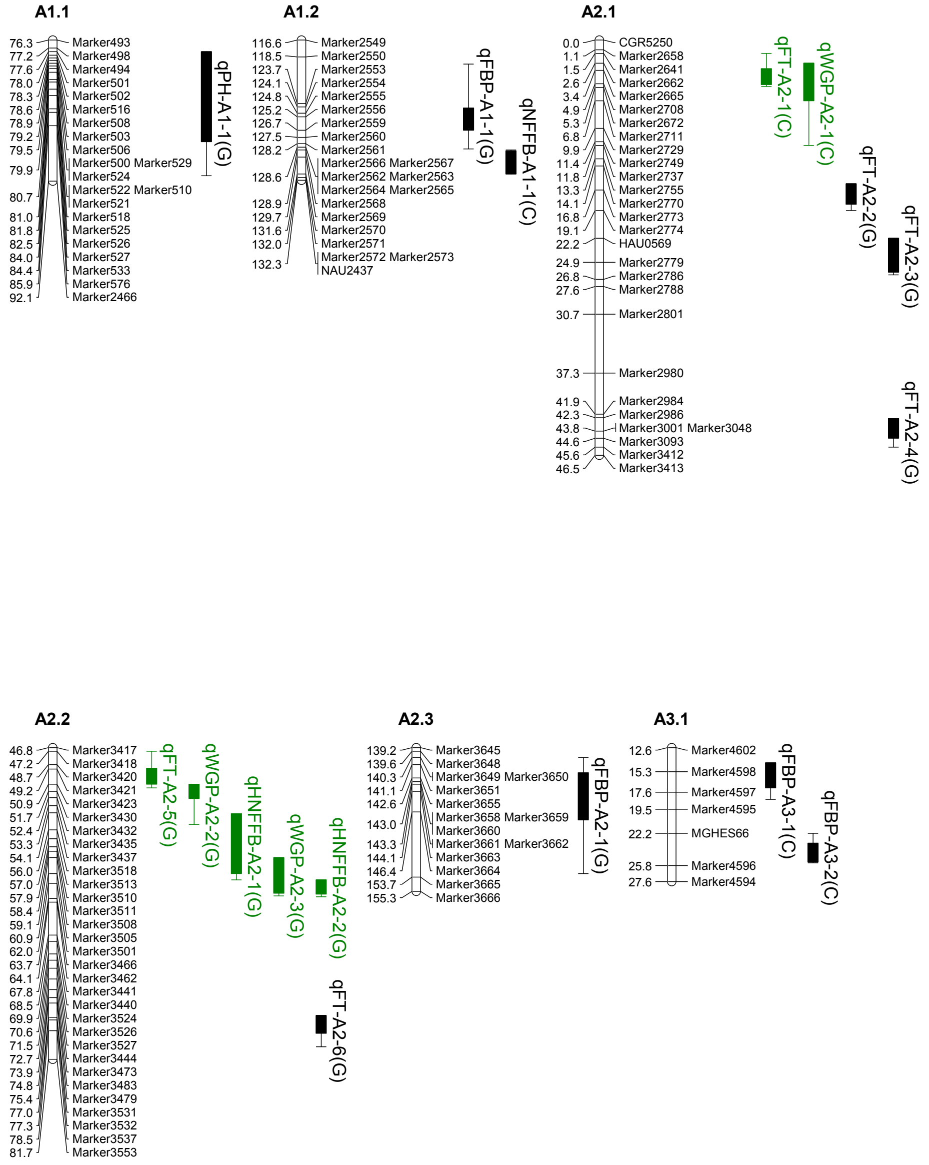

QTL distribution on the 26 chromosomes

Green QTL bars indicate QTL located in coincidence regions; black QTL bars indicate specific QTL without overlapped with other QTL; alleles conferred by CCRI36 were marked by (C); alleles conferred by G2005 were marked by (G).

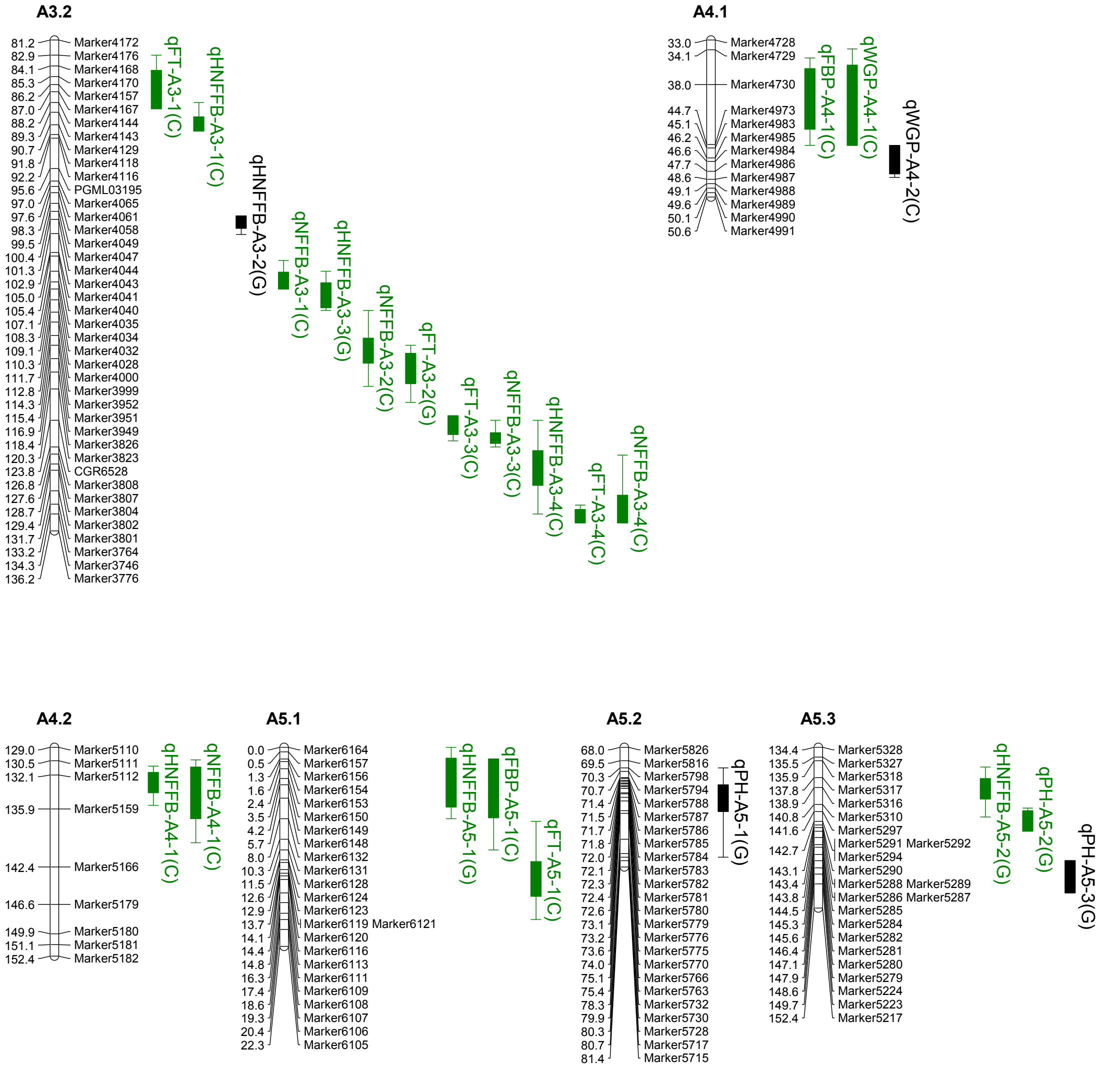
A3.2
A4.1
A4.2
A5.1
A5.2
A5.3
qFT-A3-1(C)
qHNFFB-A3-1(C)
qHNFFB-A3-2(G)
qNFFB-A3-1(C)
qHNFFB-A3-3(G)
qNFFB-A3-2(C)
qFT-A3-2(G)
qFT-A3-3(C)
qNFFB-A3-3(C)
qHNFFB-A3-4(C)
qFT-A3-4(C)
qNFFB-A3-4(C)
qFBP-A4-1(C)
qWGP-A4-1(C)
qWGP-A4-2(C)
qHNFFB-A4-1(C)
qNFFB-A4-1(C)
qHNFFB-A5-1(G)
qFBP-A5-1(C)
qFT-A5-1(C)
qPH-A5-1(G)
qHNFFB-A5-2(G)
qPH-A5-2(G)
qPH-A5-3(G)

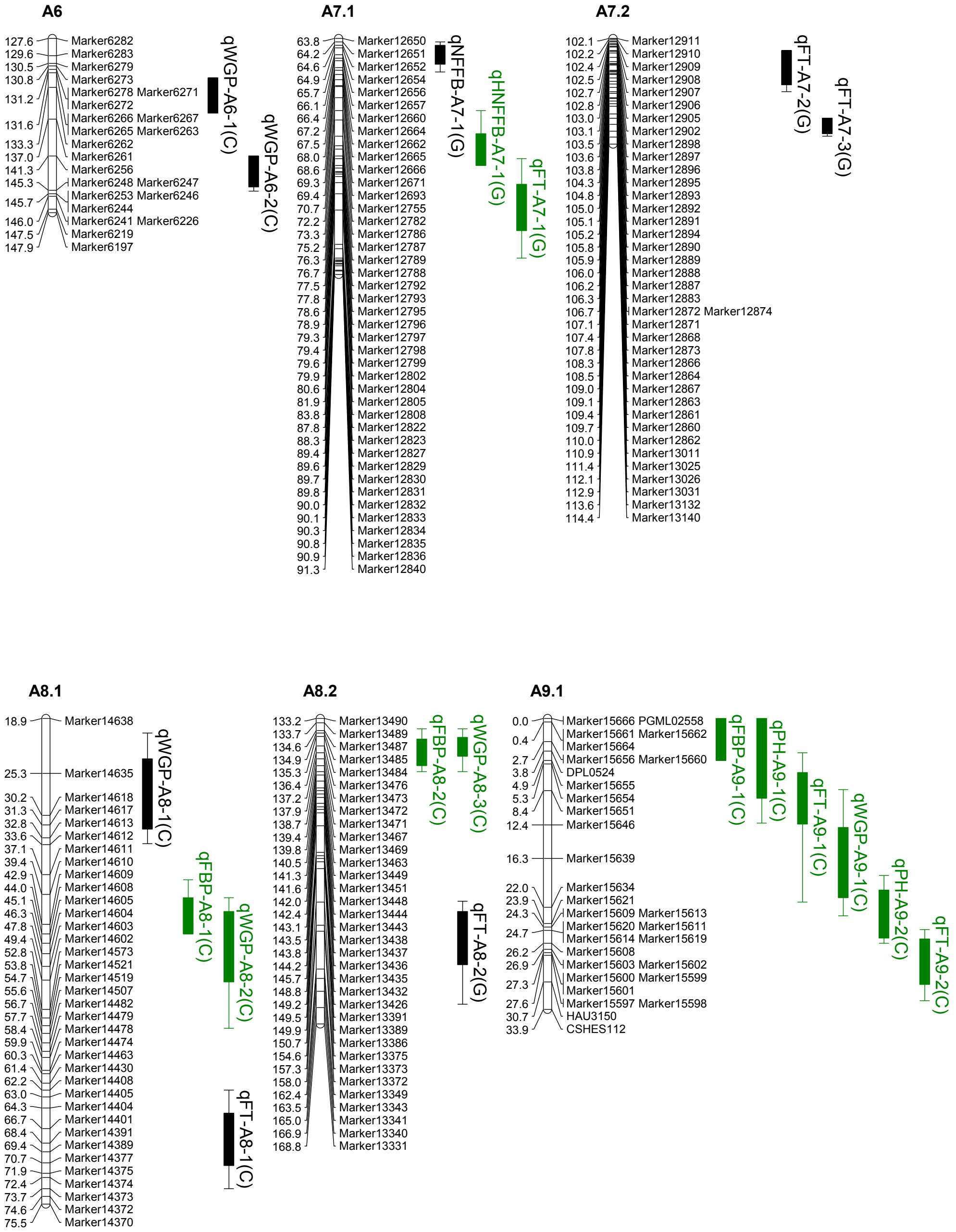
A6
127.6 Marker6282
129.6 Marker6283
130.5 Marker6279
130.8 Marker6273
131.2 Marker6278 Marker6271
Marker6272
131.6 Marker6266 Marker6267
Marker6265 Marker6263
133.3 Marker6262
137.0 Marker6261
141.3 Marker6256
145.3 Marker6248 Marker6247
145.7 Marker6253 Marker6246
Marker6244
146.0 Marker6241 Marker6226
147.5 Marker6219
147.9 Marker6197
qWGP-A6-1(C)
qWGP-A6-2(C)
A7.1
63.8 Marker12650
64.2 Marker12651
64.6 Marker12652
64.9 Marker12654
65.7 Marker12656
66.1 Marker12657
66.4 Marker12660
67.2 Marker12664
67.5 Marker12662
68.0 Marker12665
68.6 Marker12666
69.3 Marker12671
69.4 Marker12693
70.7 Marker12755
72.2 Marker12782
73.3 Marker12786
75.2 Marker12787
76.3 Marker12789
76.7 Marker12788
77.5 Marker12792
77.8 Marker12793
78.6 Marker12795
78.9 Marker12796
79.3 Marker12797
79.4 Marker12798
79.6 Marker12799
79.9 Marker12802
80.6 Marker12804
81.9 Marker12805
83.8 Marker12808
87.8 Marker12822
88.3 Marker12823
89.4 Marker12827
89.6 Marker12829
89.7 Marker12830
89.8 Marker12831
90.0 Marker12832
90.1 Marker12833
90.3 Marker12834
90.8 Marker12835
90.9 Marker12836
91.3 Marker12840
qNFFB-A7-1(G)
qHNFFB-A7-1(G)
qFT-A7-1(G)
A7.2
102.1 Marker12911
102.2 Marker12910
102.4 Marker12909
102.5 Marker12908
102.7 Marker12907
102.8 Marker12906
103.0 Marker12905
103.1 Marker12902
103.5 Marker12898
103.6 Marker12897
103.8 Marker12896
104.3 Marker12895
104.8 Marker12893
105.0 Marker12892
105.1 Marker12891
105.2 Marker12894
105.8 Marker12890
105.9 Marker12889
106.0 Marker12888
106.2 Marker12887
106.3 Marker12883
106.7 Marker12872 Marker12874
107.1 Marker12871
107.4 Marker12868
107.8 Marker12873
108.3 Marker12866
108.5 Marker12864
109.0 Marker12867
109.1 Marker12863
109.4 Marker12861
109.7 Marker12860
110.0 Marker12862
110.9 Marker13011
111.4 Marker13025
112.1 Marker13026
112.9 Marker13031
113.6 Marker13132
114.4 Marker13140
qFT-A7-2(G)
qFT-A7-3(G)
A8.1
18.9 Marker14638
25.3 Marker14635
30.2 Marker14618
31.3 Marker14617
32.8 Marker14613
33.6 Marker14612
37.1 Marker14611
39.4 Marker14610
42.9 Marker14609
44.0 Marker14608
45.1 Marker14605
46.3 Marker14604
47.8 Marker14603
49.4 Marker14602
52.8 Marker14573
53.8 Marker14521
54.7 Marker14519
55.6 Marker14507
56.7 Marker14482
57.7 Marker14479
58.4 Marker14478
59.9 Marker14474
60.3 Marker14463
61.4 Marker14430
62.2 Marker14408
63.0 Marker14405
64.3 Marker14404
66.7 Marker14401
68.4 Marker14391
69.4 Marker14389
70.7 Marker14377
71.9 Marker14375
72.4 Marker14374
73.7 Marker14373
74.6 Marker14372
75.5 Marker14370
qWGP-A8-1(C)
qFBP-A8-1(C)
qWGP-A8-2(C)
qFT-A8-1(C)
A8.2
133.2 Marker13490
133.7 Marker13489
134.6 Marker13487
134.9 Marker13485
135.3 Marker13484
136.4 Marker13476
137.2 Marker13473
137.9 Marker13472
138.7 Marker13471
139.4 Marker13467
139.8 Marker13469
140.5 Marker13463
141.3 Marker13449
141.6 Marker13451
142.0 Marker13448
142.4 Marker13444
143.1 Marker13443
143.5 Marker13438
143.8 Marker13437
144.2 Marker13436
145.7 Marker13435
148.8 Marker13432
149.2 Marker13426
149.5 Marker13391
149.9 Marker13389
150.7 Marker13386
154.6 Marker13375
157.3 Marker13373
158.0 Marker13372
162.4 Marker13349
163.5 Marker13343
165.0 Marker13341
166.9 Marker13340
168.8 Marker13331
qFBP-A8-2(C)
qWGP-A8-3(C)
qFT-A8-2(G)
A9.1
0.0 Marker15666 PGML02558
0.4 Marker15661 Marker15662
Marker15664
2.7 Marker15656 Marker15660
3.8 DPL0524
4.9 Marker15655
5.3 Marker15654
8.4 Marker15651
12.4 Marker15646
16.3 Marker15639
22.0 Marker15634
23.9 Marker15621
24.3 Marker15609 Marker15613
24.7 Marker15620 Marker15611
Marker15614 Marker15619
26.2 Marker15608
26.9 Marker15603 Marker15602
27.3 Marker15600 Marker15599
Marker15601
27.6 Marker15597 Marker15598
30.7 HAU3150
33.9 CSHES112
qFBP-A9-1(C)
qPH-A9-1(C)
qFT-A9-1(C)
qWGP-A9-1(C)
qPH-A9-2(C)
qFT-A9-2(C)

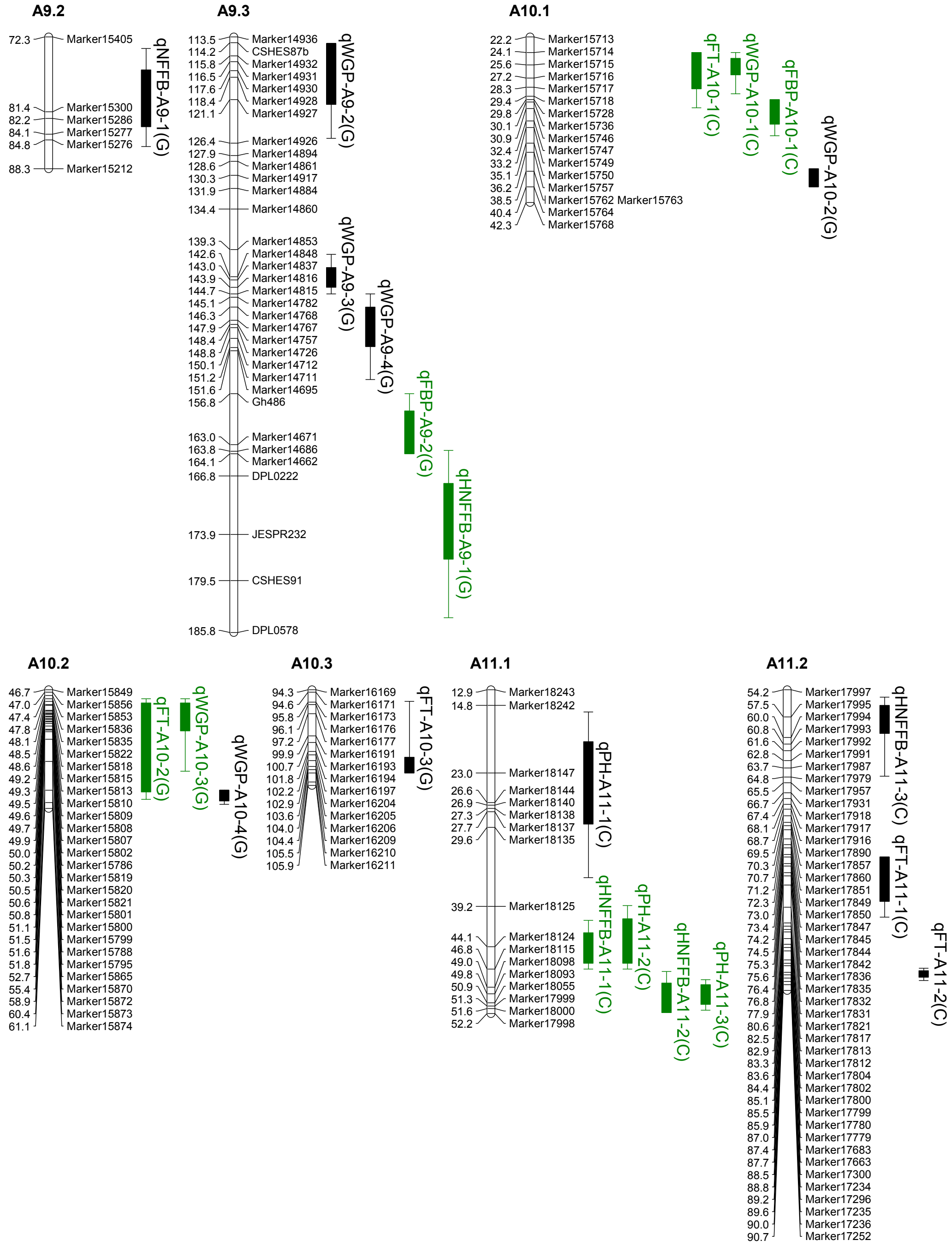
A9.2
72.3 Marker15405
81.4 Marker15300
82.2 Marker15286
84.1 Marker15277
84.8 Marker15276
88.3 Marker15212
qNFFB-A9-1(G)
A9.3
113.5 Marker14936
114.2 CSHES87b
115.8 Marker14932
116.5 Marker14931
117.6 Marker14930
118.4 Marker14928
121.1 Marker14927
126.4 Marker14926
127.9 Marker14894
128.6 Marker14861
130.3 Marker14917
131.9 Marker14884
134.4 Marker14860
139.3 Marker14853
142.6 Marker14848
143.0 Marker14837
143.9 Marker14816
144.7 Marker14815
145.1 Marker14782
146.3 Marker14768
147.9 Marker14767
148.4 Marker14757
148.8 Marker14726
150.1 Marker14712
151.2 Marker14711
151.6 Marker14695
156.8 Gh486
163.0 Marker14671
163.8 Marker14686
164.1 Marker14662
166.8 DPL0222
173.9 JESPR232
179.5 CSHES91
185.8 DPL0578
qWGP-A9-2(G)
qWGP-A9-3(G)
qWGP-A9-4(G)
qFBP-A9-2(G)
qHNFFB-A9-1(G)
A10.1
22.2 Marker15713
24.1 Marker15714
25.6 Marker15715
27.2 Marker15716
28.3 Marker15717
29.4 Marker15718
29.8 Marker15728
30.1 Marker15736
30.9 Marker15746
32.4 Marker15747
33.2 Marker15749
35.1 Marker15750
36.2 Marker15757
38.5 Marker15762 Marker15763
40.4 Marker15764
42.3 Marker15768
qFT-A10-1(C)
qWGP-A10-1(C)
qFBP-A10-1(C)
qWGP-A10-2(G)
A10.2
46.7 Marker15849
47.0 Marker15856
47.4 Marker15853
47.8 Marker15836
48.1 Marker15835
48.5 Marker15822
48.6 Marker15818
49.2 Marker15815
49.3 Marker15813
49.5 Marker15810
49.6 Marker15809
49.7 Marker15808
49.9 Marker15807
50.0 Marker15802
50.2 Marker15786
50.3 Marker15819
50.5 Marker15820
50.6 Marker15821
50.8 Marker15801
51.1 Marker15800
51.5 Marker15799
51.6 Marker15788
51.8 Marker15795
52.7 Marker15865
55.4 Marker15870
58.9 Marker15872
60.4 Marker15873
61.1 Marker15874
qFT-A10-2(G)
qWGP-A10-3(G)
qWGP-A10-4(G)
A10.3
94.3 Marker16169
94.6 Marker16171
95.8 Marker16173
96.1 Marker16176
97.2 Marker16177
99.9 Marker16191
100.7 Marker16193
101.8 Marker16194
102.2 Marker16197
102.9 Marker16204
103.6 Marker16205
104.0 Marker16206
104.4 Marker16209
105.5 Marker16210
105.9 Marker16211
qFT-A10-3(G)
A11.1
12.9 Marker18243
14.8 Marker18242
23.0 Marker18147
26.6 Marker18144
26.9 Marker18140
27.3 Marker18138
27.7 Marker18137
29.6 Marker18135
39.2 Marker18125
44.1 Marker18124
46.8 Marker18115
49.0 Marker18098
49.8 Marker18093
50.9 Marker18055
51.3 Marker17999
51.6 Marker18000
52.2 Marker17998
qPH-A11-1(C)
qHNFFB-A11-1(C)
qPH-A11-2(C)
qHNFFB-A11-2(C)
qPH-A11-3(C)
A11.2
54.2 Marker17997
57.5 Marker17995
60.0 Marker17994
60.8 Marker17993
61.6 Marker17992
62.8 Marker17991
63.7 Marker17987
64.8 Marker17979
65.5 Marker17957
66.7 Marker17931
67.4 Marker17918
68.1 Marker17917
68.7 Marker17916
69.5 Marker17890
70.3 Marker17857
70.7 Marker17860
71.2 Marker17851
72.3 Marker17849
73.0 Marker17850
73.4 Marker17847
74.2 Marker17845
74.5 Marker17844
75.3 Marker17842
75.6 Marker17836
76.4 Marker17835
76.8 Marker17832
77.9 Marker17831
80.6 Marker17821
82.5 Marker17817
82.9 Marker17813
83.3 Marker17812
83.6 Marker17804
84.4 Marker17802
85.1 Marker17800
85.5 Marker17799
85.9 Marker17780
87.0 Marker17779
87.4 Marker17683
87.7 Marker17663
88.5 Marker17300
88.8 Marker17234
89.2 Marker17296
89.6 Marker17235
90.0 Marker17236
90.7 Marker17252
qHNFFB-A11-3(C)
qFT-A11-1(C)
qFT-A11-2(C)

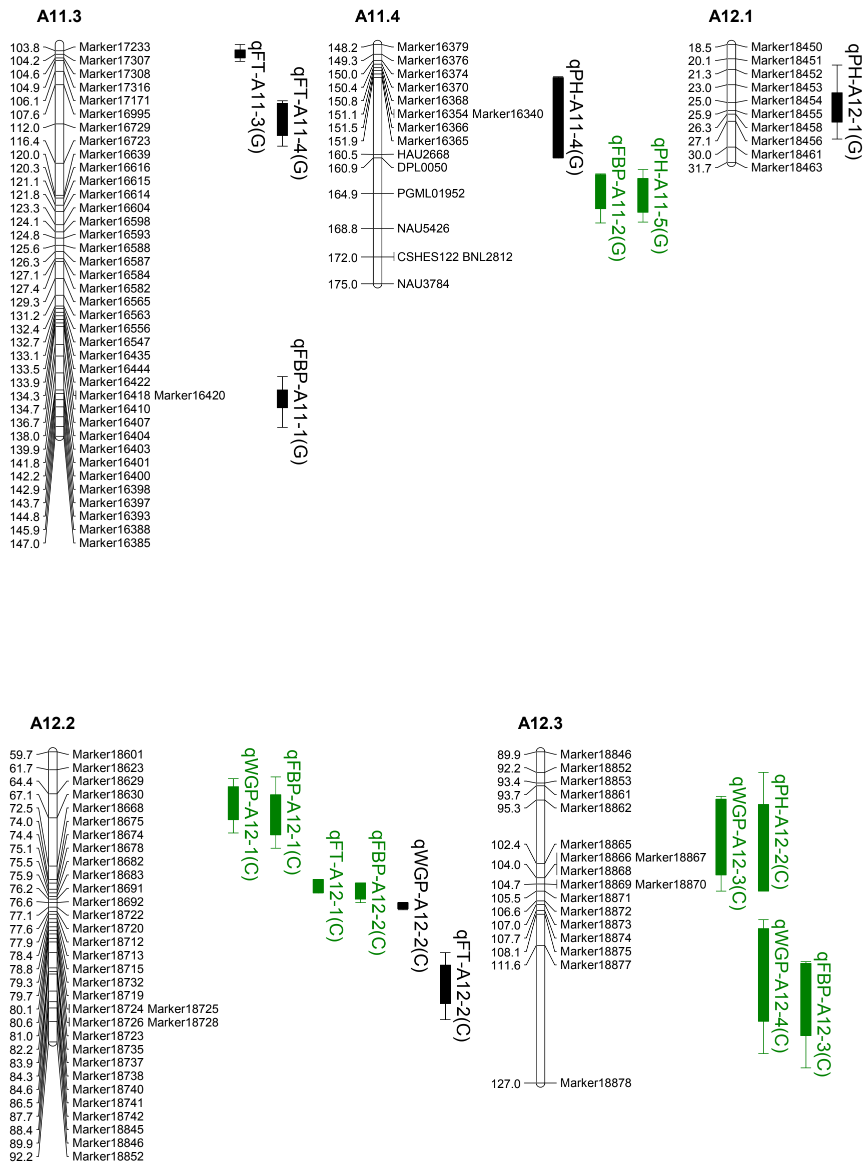
A11.3
A11.4
A12.1
A12.2
A12.3
qFT-A11-3(G)
qFT-A11-4(G)
qFBP-A11-1(G)
qPH-A11-4(G)
qFBP-A11-2(G)
qPH-A11-5(G)
qPH-A12-1(G)
qWGP-A12-1(C)
qFBP-A12-1(C)
qFT-A12-1(C)
qFBP-A12-2(C)
qWGP-A12-2(C)
qFT-A12-2(C)
qWGP-A12-3(C)
qPH-A12-2(C)
qWGP-A12-4(C)
qFBP-A12-3(C)

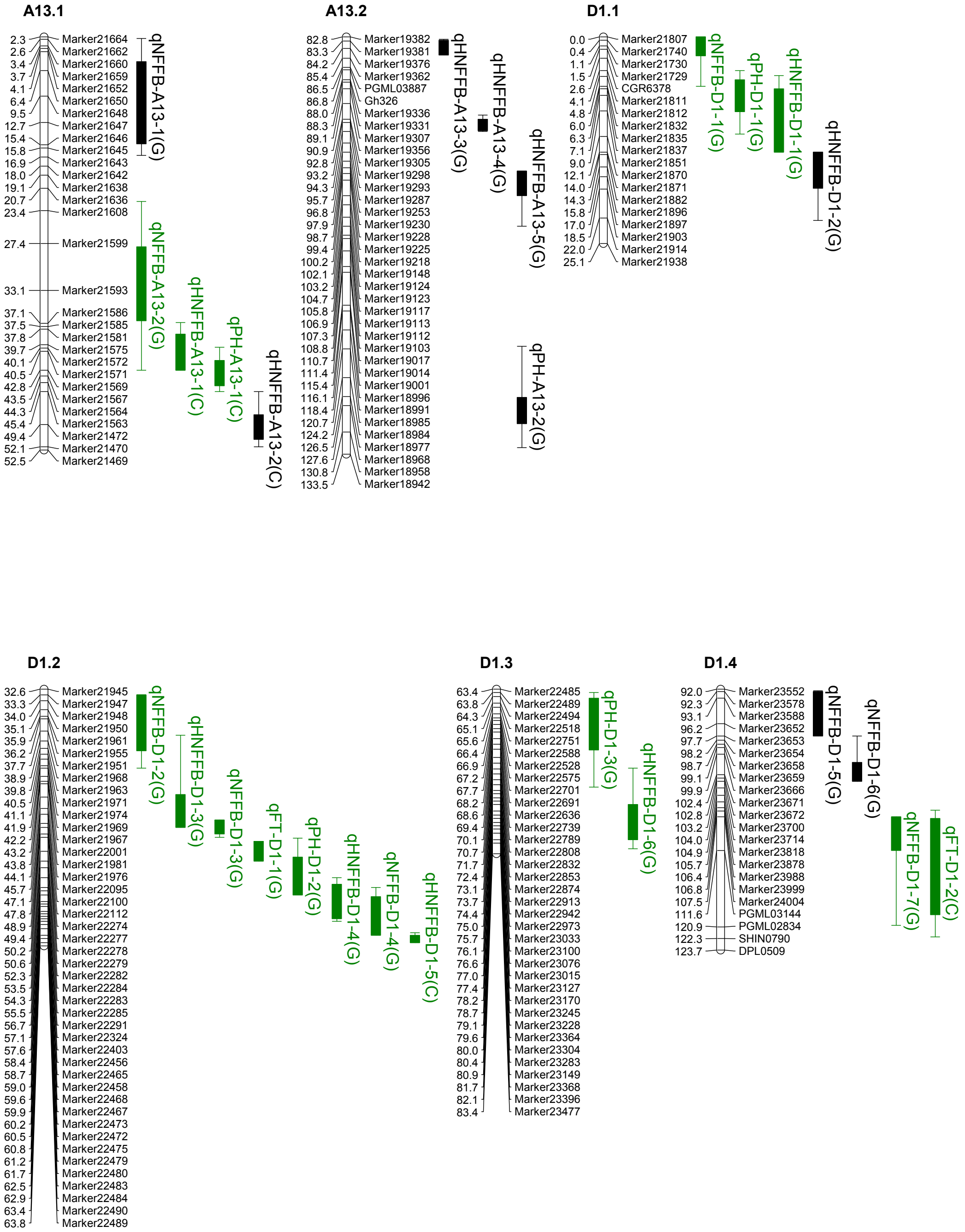
A13.1
2.3 Marker21664
2.6 Marker21662
3.4 Marker21660
3.7 Marker21659
4.1 Marker21652
6.4 Marker21650
9.5 Marker21648
12.7 Marker21647
15.4 Marker21646
15.8 Marker21645
16.9 Marker21643
18.0 Marker21642
19.1 Marker21638
20.7 Marker21636
23.4 Marker21608
27.4 Marker21599
33.1 Marker21593
37.1 Marker21586
37.5 Marker21585
37.8 Marker21581
39.7 Marker21575
40.1 Marker21572
40.5 Marker21571
42.8 Marker21569
43.5 Marker21567
44.3 Marker21564
45.4 Marker21563
49.4 Marker21472
52.1 Marker21470
52.5 Marker21469
qNFFB-A13-1(G)
qNFFB-A13-2(G)
qHNFFB-A13-1(C)
qPH-A13-1(C)
qHNFFB-A13-2(C)
A13.2
82.8 Marker19382
83.3 Marker19381
84.2 Marker19376
85.4 Marker19362
86.5 PGML03887
86.8 Gh326
88.0 Marker19336
88.3 Marker19331
89.1 Marker19307
90.9 Marker19356
92.8 Marker19305
93.2 Marker19298
94.3 Marker19293
95.7 Marker19287
96.8 Marker19253
97.9 Marker19230
98.7 Marker19228
99.4 Marker19225
100.2 Marker19218
102.1 Marker19148
103.2 Marker19124
104.7 Marker19123
105.8 Marker19117
106.9 Marker19113
107.3 Marker19112
108.8 Marker19103
110.7 Marker19017
111.4 Marker19014
115.4 Marker19001
116.1 Marker18996
118.4 Marker18991
120.7 Marker18985
124.2 Marker18984
126.5 Marker18977
127.6 Marker18968
130.8 Marker18958
133.5 Marker18942
qHNFFB-A13-3(G)
qHNFFB-A13-4(G)
qHNFFB-A13-5(G)
qPH-A13-2(G)
D1.1
0.0 Marker21807
0.4 Marker21740
1.1 Marker21730
1.5 Marker21729
2.6 CGR6378
4.1 Marker21811
4.8 Marker21812
6.0 Marker21832
6.3 Marker21835
7.1 Marker21837
9.0 Marker21851
12.1 Marker21870
14.0 Marker21871
14.3 Marker21882
15.8 Marker21896
17.0 Marker21897
18.5 Marker21903
22.0 Marker21914
25.1 Marker21938
qNFFB-D1-1(G)
qPH-D1-1(G)
qHNFFB-D1-1(G)
qHNFFB-D1-2(G)
D1.2
32.6 Marker21945
33.3 Marker21947
34.0 Marker21948
35.1 Marker21950
35.9 Marker21961
36.2 Marker21955
37.7 Marker21951
38.9 Marker21968
39.8 Marker21963
40.5 Marker21971
41.1 Marker21974
41.9 Marker21969
42.2 Marker21967
43.2 Marker22001
43.8 Marker21981
44.1 Marker21976
45.7 Marker22095
47.1 Marker22100
47.8 Marker22112
48.9 Marker22274
49.4 Marker22277
50.2 Marker22278
50.6 Marker22279
52.3 Marker22282
53.5 Marker22284
54.3 Marker22283
55.5 Marker22285
56.7 Marker22291
57.1 Marker22324
57.6 Marker22403
58.4 Marker22456
58.7 Marker22465
59.0 Marker22458
59.6 Marker22468
59.9 Marker22467
60.2 Marker22473
60.5 Marker22472
60.8 Marker22475
61.2 Marker22479
61.7 Marker22480
62.5 Marker22483
62.9 Marker22484
63.4 Marker22490
63.8 Marker22489
qNFFB-D1-2(G)
qHNFFB-D1-3(G)
qNFFB-D1-3(G)
qFT-D1-1(G)
qPH-D1-2(G)
qHNFFB-D1-4(G)
qNFFB-D1-4(G)
qHNFFB-D1-5(C)
D1.3
63.4 Marker22485
63.8 Marker22489
64.3 Marker22494
65.1 Marker22518
65.6 Marker22751
66.4 Marker22588
66.9 Marker22528
67.2 Marker22575
67.7 Marker22701
68.2 Marker22691
68.6 Marker22636
69.4 Marker22739
70.1 Marker22789
70.7 Marker22808
71.7 Marker22832
72.4 Marker22853
73.1 Marker22874
73.7 Marker22913
74.4 Marker22942
75.0 Marker22973
75.7 Marker23033
76.1 Marker23100
76.6 Marker23076
77.0 Marker23015
77.4 Marker23127
78.2 Marker23170
78.7 Marker23245
79.1 Marker23228
79.6 Marker23364
80.0 Marker23304
80.4 Marker23283
80.9 Marker23149
81.7 Marker23368
82.1 Marker23396
83.4 Marker23477
qPH-D1-3(G)
qHNFFB-D1-6(G)
D1.4
92.0 Marker23552
92.3 Marker23578
93.1 Marker23588
96.2 Marker23652
97.7 Marker23653
98.2 Marker23654
98.7 Marker23658
99.1 Marker23659
99.9 Marker23666
102.4 Marker23671
102.8 Marker23672
103.2 Marker23700
104.0 Marker23714
104.9 Marker23818
105.7 Marker23878
106.4 Marker23988
106.8 Marker23999
107.5 Marker24004
111.6 PGML03144
120.9 PGML02834
122.3 SHIN0790
123.7 DPL0509
qNFFB-D1-5(G)
qNFFB-D1-6(G)
qNFFB-D1-7(G)
qFT-D1-2(C)

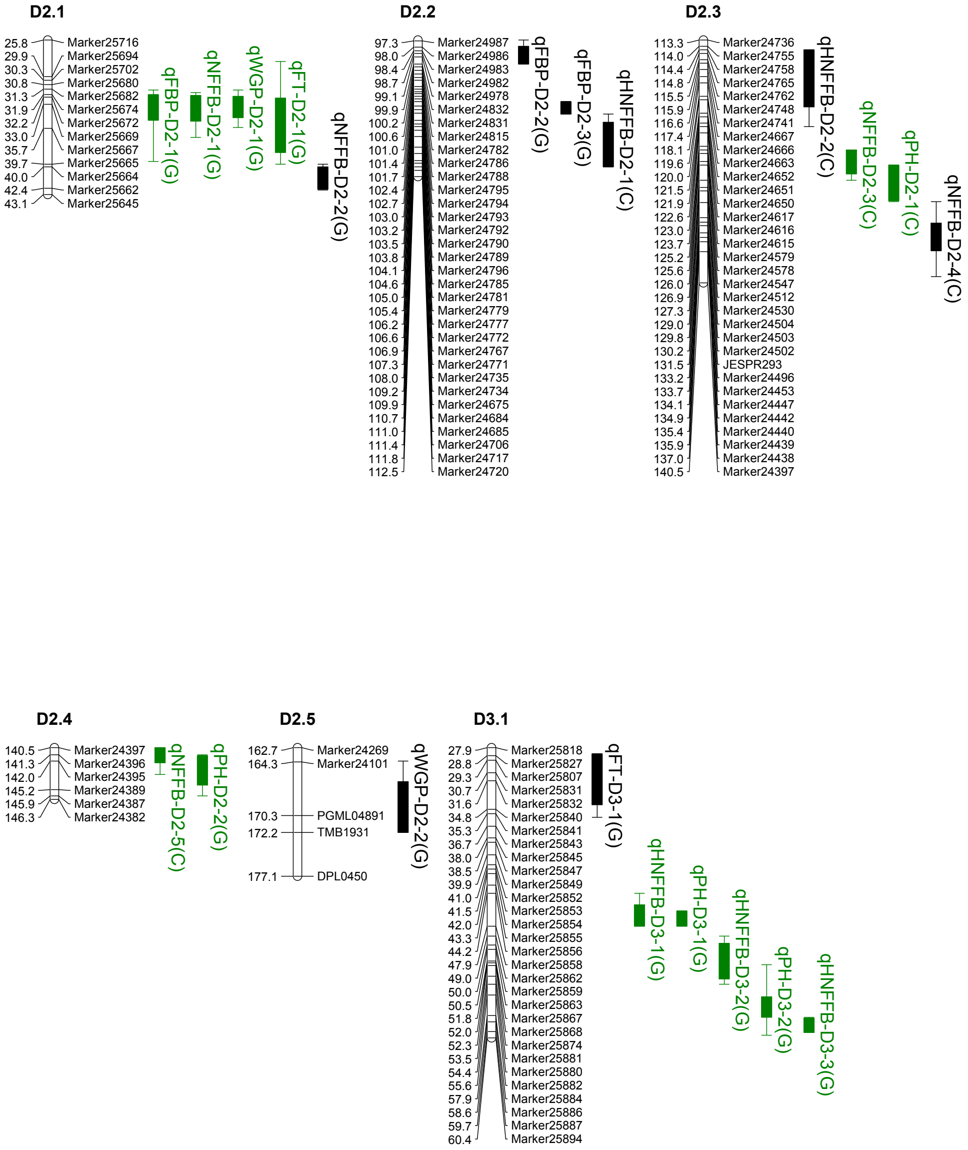
D2.1
25.8 Marker25716
29.9 Marker25694
30.3 Marker25702
30.8 Marker25680
31.3 Marker25682
31.9 Marker25674
32.2 Marker25672
33.0 Marker25669
35.7 Marker25667
39.7 Marker25665
40.0 Marker25664
42.4 Marker25662
43.1 Marker25645
qFBP-D2-1(G)
qNFFB-D2-1(G)
qWGP-D2-1(G)
qFT-D2-1(G)
qNFFB-D2-2(G)
D2.2
97.3 Marker24987
98.0 Marker24986
98.4 Marker24983
98.7 Marker24982
99.1 Marker24978
99.9 Marker24832
100.2 Marker24831
100.6 Marker24815
101.0 Marker24782
101.4 Marker24786
101.7 Marker24788
102.4 Marker24795
102.7 Marker24794
103.0 Marker24793
103.2 Marker24792
103.5 Marker24790
103.8 Marker24789
104.1 Marker24796
104.6 Marker24785
105.0 Marker24781
105.4 Marker24779
106.2 Marker24777
106.6 Marker24772
106.9 Marker24767
107.3 Marker24771
108.0 Marker24735
109.2 Marker24734
109.9 Marker24675
110.7 Marker24684
111.0 Marker24685
111.4 Marker24706
111.8 Marker24717
112.5 Marker24720
qFBP-D2-2(G)
qFBP-D2-3(G)
qHNFFB-D2-1(C)
D2.3
113.3 Marker24736
114.0 Marker24755
114.4 Marker24758
114.8 Marker24765
115.5 Marker24762
115.9 Marker24748
116.6 Marker24741
117.4 Marker24667
118.1 Marker24666
119.6 Marker24663
120.0 Marker24652
121.5 Marker24651
121.9 Marker24650
122.6 Marker24617
123.0 Marker24616
123.7 Marker24615
125.2 Marker24579
125.6 Marker24578
126.0 Marker24547
126.9 Marker24512
127.3 Marker24530
129.0 Marker24504
129.8 Marker24503
130.2 Marker24502
131.5 JESPR293
133.2 Marker24496
133.7 Marker24453
134.1 Marker24447
134.9 Marker24442
135.4 Marker24440
135.9 Marker24439
137.0 Marker24438
140.5 Marker24397
qHNFFB-D2-2(C)
qNFFB-D2-3(C)
qPH-D2-1(C)
qNFFB-D2-4(C)
D2.4
140.5 Marker24397
141.3 Marker24396
142.0 Marker24395
145.2 Marker24389
145.9 Marker24387
146.3 Marker24382
qNFFB-D2-5(C)
qPH-D2-2(G)
D2.5
162.7 Marker24269
164.3 Marker24101
170.3 PGML04891
172.2 TMB1931
177.1 DPL0450
qWGP-D2-2(G)
D3.1
27.9 Marker25818
28.8 Marker25827
29.3 Marker25807
30.7 Marker25831
31.6 Marker25832
34.8 Marker25840
35.3 Marker25841
36.7 Marker25843
38.0 Marker25845
38.5 Marker25847
39.9 Marker25849
41.0 Marker25852
41.5 Marker25853
42.0 Marker25854
43.3 Marker25855
44.2 Marker25856
47.9 Marker25858
49.0 Marker25862
50.0 Marker25859
50.5 Marker25863
51.8 Marker25867
52.0 Marker25868
52.3 Marker25874
53.5 Marker25881
54.4 Marker25880
55.6 Marker25882
57.9 Marker25884
58.6 Marker25886
59.7 Marker25887
60.4 Marker25894
qFT-D3-1(G)
qHNFFB-D3-1(G)
qPH-D3-1(G)
qHNFFB-D3-2(G)
qPH-D3-2(G)
qHNFFB-D3-3(G)

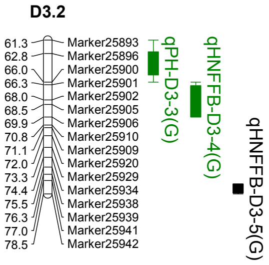
D3.2
61.3
62.8
66.0
66.3
68.0
68.5
69.9
70.8
71.1
72.0
73.3
74.4
75.5
76.3
77.0
78.5
Marker25893
Marker25896
Marker25900
Marker25901
Marker25902
Marker25905
Marker25906
Marker25910
Marker25909
Marker25920
Marker25929
Marker25934
Marker25938
Marker25939
Marker25941
Marker25942
qPH-D3-3(G)
qHNFFB-D3-4(G)
qHNFFB-D3-5(G)

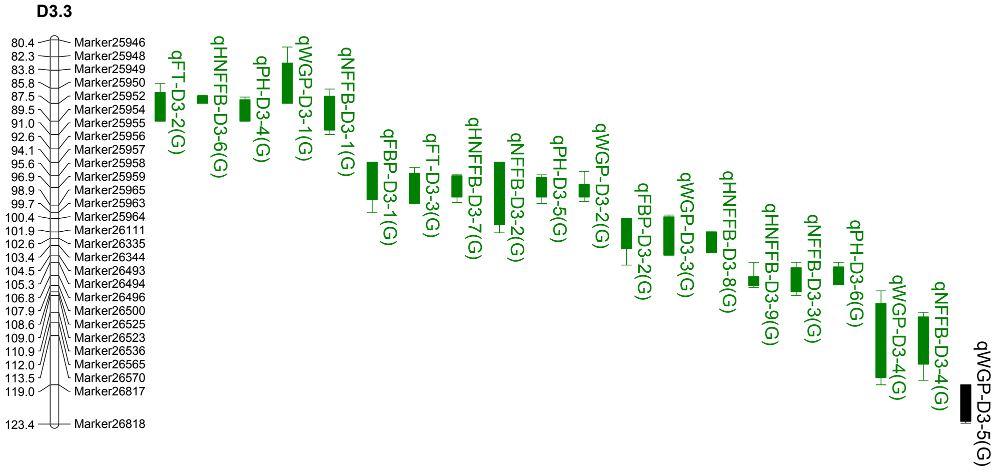
D3.3
80.4
82.3
83.8
85.8
87.5
89.5
91.0
92.6
94.1
95.6
96.9
98.9
99.7
100.4
101.9
102.6
103.4
104.5
105.3
106.8
107.9
108.6
109.0
110.9
112.0
113.5
119.0
123.4
Marker25946
Marker25948
Marker25949
Marker25950
Marker25952
Marker25954
Marker25955
Marker25956
Marker25957
Marker25958
Marker25959
Marker25965
Marker25963
Marker25964
Marker26111
Marker26335
Marker26344
Marker26493
Marker26494
Marker26496
Marker26500
Marker26525
Marker26523
Marker26536
Marker26565
Marker26570
Marker26817
Marker26818
qFT-D3-2(G)
qHNFFB-D3-6(G)
qPH-D3-4(G)
qWGP-D3-1(G)
qNFFB-D3-1(G)
qFBP-D3-1(G)
qFT-D3-3(G)
qHNFFB-D3-7(G)
qNFFB-D3-2(G)
qPH-D3-5(G)
qWGP-D3-2(G)
qFBP-D3-2(G)
qWGP-D3-3(G)
qHNFFB-D3-8(G)
qHNFFB-D3-9(G)
qNFFB-D3-3(G)
qPH-D3-6(G)
qWGP-D3-4(G)
qNFFB-D3-4(G)
qWGP-D3-5(G)

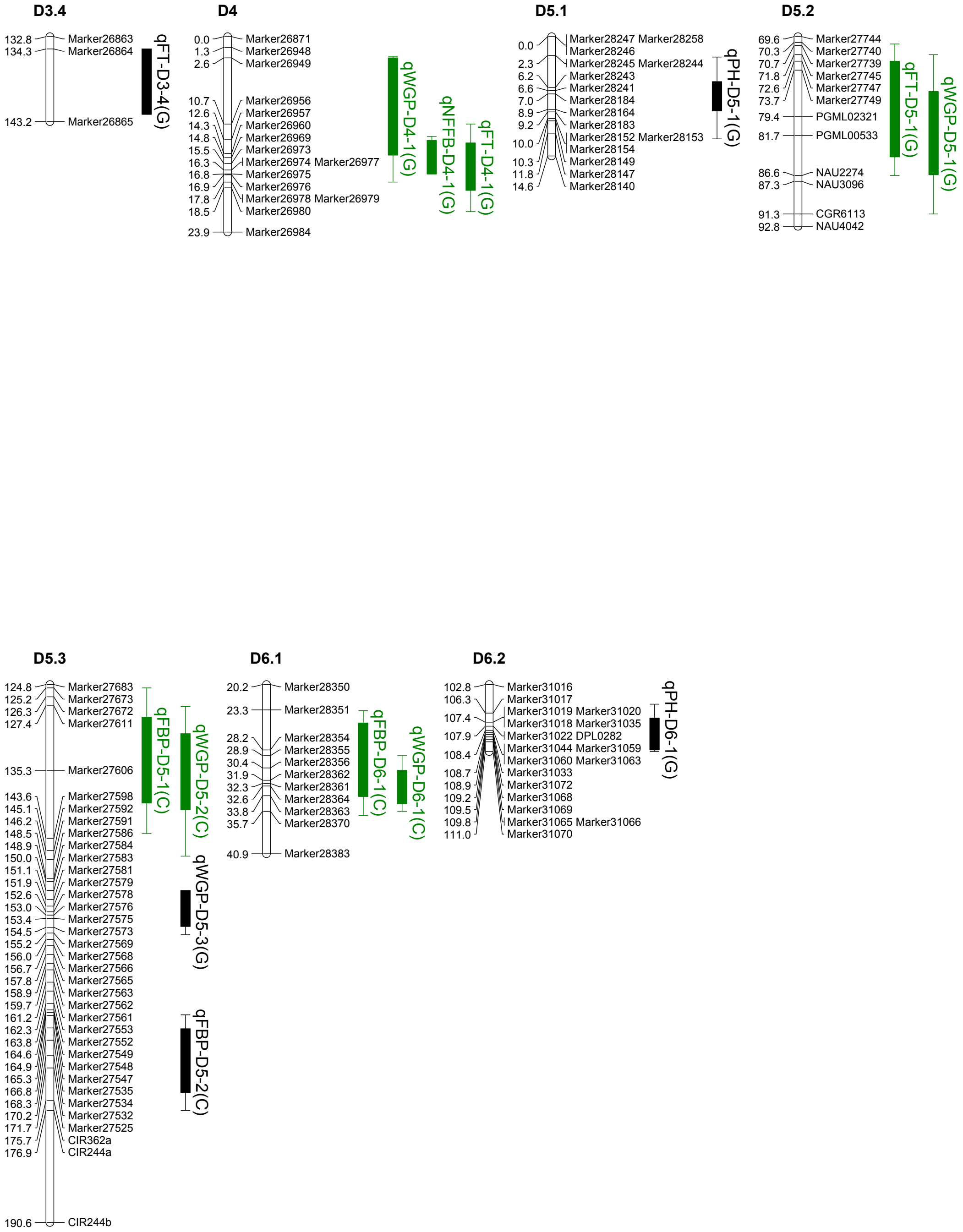
D3.4
132.8 Marker26863
134.3 Marker26864
143.2 Marker26865
qFT-D3-4(G)
D4
0.0 Marker26871
1.3 Marker26948
2.6 Marker26949
10.7 Marker26956
12.6 Marker26957
14.3 Marker26960
14.8 Marker26969
15.5 Marker26973
16.3 Marker26974 Marker26977
16.8 Marker26975
16.9 Marker26976
17.8 Marker26978 Marker26979
18.5 Marker26980
23.9 Marker26984
qWGP-D4-1(G)
qNFFB-D4-1(G)
qFT-D4-1(G)
D5.1
0.0 Marker28247 Marker28258
Marker28246
2.3 Marker28245 Marker28244
6.2 Marker28243
6.6 Marker28241
7.0 Marker28184
8.9 Marker28164
9.2 Marker28183
10.0 Marker28152 Marker28153
Marker28154
10.3 Marker28149
11.8 Marker28147
14.6 Marker28140
qPH-D5-1(G)
D5.2
69.6 Marker27744
70.3 Marker27740
70.7 Marker27739
71.8 Marker27745
72.6 Marker27747
73.7 Marker27749
79.4 PGML02321
81.7 PGML00533
86.6 NAU2274
87.3 NAU3096
91.3 CGR6113
92.8 NAU4042
qFT-D5-1(G)
qWGP-D5-1(G)
D5.3
124.8 Marker27683
125.2 Marker27673
126.3 Marker27672
127.4 Marker27611
135.3 Marker27606
143.6 Marker27598
145.1 Marker27592
146.2 Marker27591
148.5 Marker27586
148.9 Marker27584
150.0 Marker27583
151.1 Marker27581
151.9 Marker27579
152.6 Marker27578
153.0 Marker27576
153.4 Marker27575
154.5 Marker27573
155.2 Marker27569
156.0 Marker27568
156.7 Marker27566
157.8 Marker27565
158.9 Marker27563
159.7 Marker27562
161.2 Marker27561
162.3 Marker27553
163.8 Marker27552
164.6 Marker27549
164.9 Marker27548
165.3 Marker27547
166.8 Marker27535
168.3 Marker27534
170.2 Marker27532
171.7 Marker27525
175.7 CIR362a
176.9 CIR244a
190.6 CIR244b
qFBP-D5-1(C)
qWGP-D5-2(C)
qWGP-D5-3(G)
qFBP-D5-2(C)
D6.1
20.2 Marker28350
23.3 Marker28351
28.2 Marker28354
28.9 Marker28355
30.4 Marker28356
31.9 Marker28362
32.3 Marker28361
32.6 Marker28364
33.8 Marker28363
35.7 Marker28370
40.9 Marker28383
qFBP-D6-1(C)
qWGP-D6-1(C)
D6.2
102.8 Marker31016
106.3 Marker31017
107.4 Marker31019 Marker31020
Marker31018 Marker31035
107.9 Marker31022 DPL0282
108.4 Marker31044 Marker31059
Marker31060 Marker31063
108.7 Marker31033
108.9 Marker31072
109.2 Marker31068
109.5 Marker31069
109.8 Marker31065 Marker31066
111.0 Marker31070
qPH-D6-1(G)

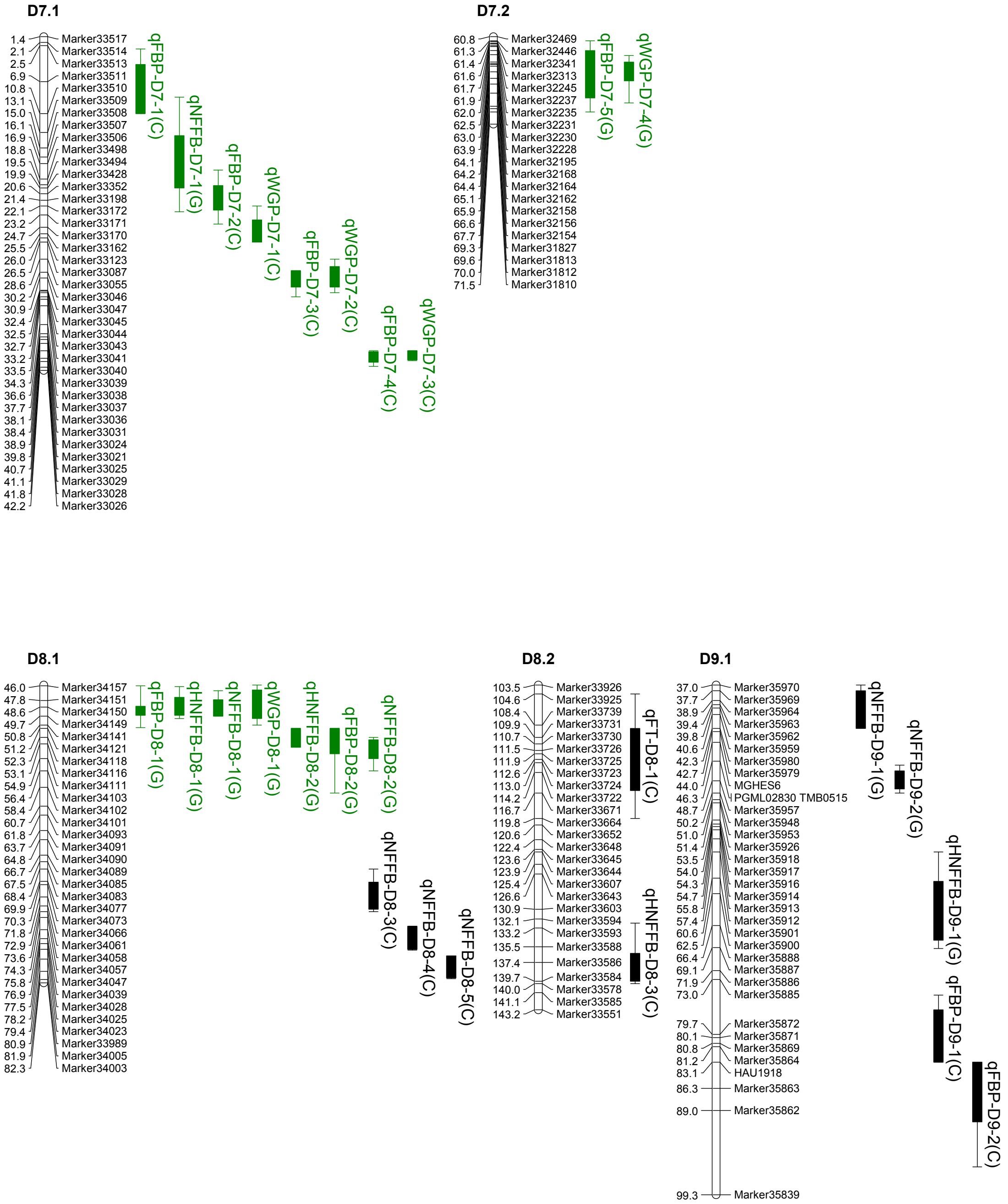

D7.1
D7.2
D8.1
D8.2
D9.1
qFBP-D7-1(C)
qNFFB-D7-1(G)
qFBP-D7-2(C)
qWGP-D7-1(C)
qFBP-D7-3(C)
qWGP-D7-2(C)
qFBP-D7-4(C)
qWGP-D7-3(C)
qFBP-D7-5(G)
qWGP-D7-4(G)
qFBP-D8-1(G)
qHNFFB-D8-1(G)
qNFFB-D8-1(G)
qWGP-D8-1(G)
qHNFFB-D8-2(G)
qFBP-D8-2(G)
qNFFB-D8-2(G)
qNFFB-D8-3(C)
qNFFB-D8-4(C)
qNFFB-D8-5(C)
qFT-D8-1(C)
qHNFFB-D8-3(C)
qNFFB-D9-1(G)
qNFFB-D9-2(G)
qHNFFB-D9-1(G)
qFBP-D9-1(C)
qFBP-D9-2(C)

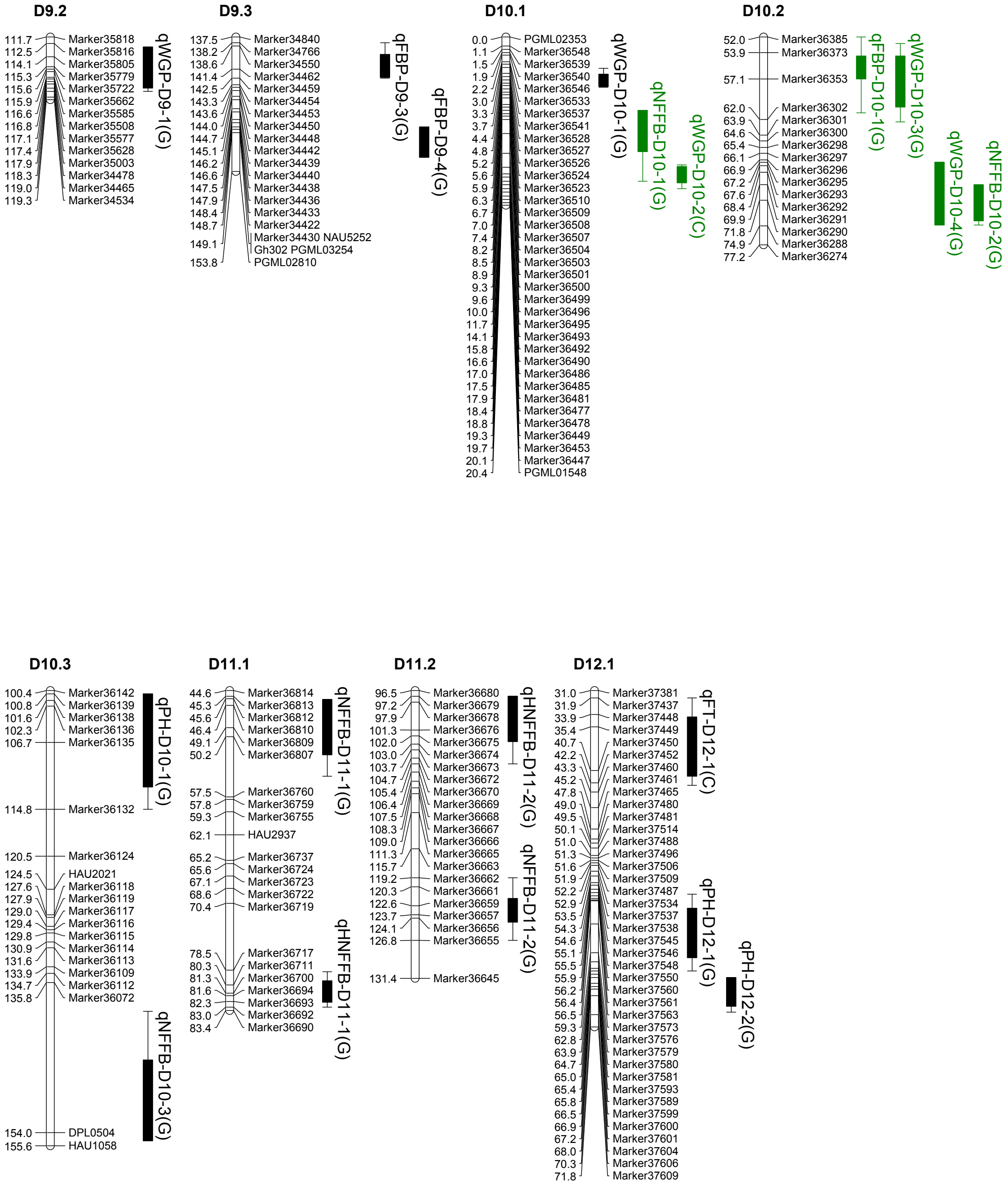
D9.2
111.7 Marker35818
112.5 Marker35816
114.1 Marker35805
115.3 Marker35779
115.6 Marker35722
115.9 Marker35662
116.6 Marker35585
116.8 Marker35508
117.1 Marker35577
117.4 Marker35628
117.9 Marker35003
118.3 Marker34478
119.0 Marker34465
119.3 Marker34534
qWGP-D9-1(G)
D9.3
137.5 Marker34840
138.2 Marker34766
138.6 Marker34550
141.4 Marker34462
142.5 Marker34459
143.3 Marker34454
143.6 Marker34453
144.0 Marker34450
144.7 Marker34448
145.1 Marker34442
146.2 Marker34439
146.6 Marker34440
147.5 Marker34438
147.9 Marker34436
148.4 Marker34433
148.7 Marker34422
149.1 Marker34430 NAU5252 Gh302 PGML03254
153.8 PGML02810
qFBP-D9-3(G)
qFBP-D9-4(G)
D10.1
0.0 PGML02353
1.1 Marker36548
1.5 Marker36539
1.9 Marker36540
2.2 Marker36546
3.0 Marker36533
3.3 Marker36537
3.7 Marker36541
4.4 Marker36528
4.8 Marker36527
5.2 Marker36526
5.6 Marker36524
5.9 Marker36523
6.3 Marker36510
6.7 Marker36509
7.0 Marker36508
7.4 Marker36507
8.2 Marker36504
8.5 Marker36503
8.9 Marker36501
9.3 Marker36500
9.6 Marker36499
10.0 Marker36496
11.7 Marker36495
14.1 Marker36493
15.8 Marker36492
16.6 Marker36490
17.0 Marker36486
17.5 Marker36485
17.9 Marker36481
18.4 Marker36477
18.8 Marker36478
19.3 Marker36449
19.7 Marker36453
20.1 Marker36447
20.4 PGML01548
qWGP-D10-1(G)
qNFFB-D10-1(G)
qWGP-D10-2(C)
D10.2
52.0 Marker36385
53.9 Marker36373
57.1 Marker36353
62.0 Marker36302
63.9 Marker36301
64.6 Marker36300
65.4 Marker36298
66.1 Marker36297
66.9 Marker36296
67.2 Marker36295
67.6 Marker36293
68.4 Marker36292
69.9 Marker36291
71.8 Marker36290
74.9 Marker36288
77.2 Marker36274
qFBP-D10-1(G)
qWGP-D10-3(G)
qWGP-D10-4(G)
qNFFB-D10-2(G)
D10.3
100.4 Marker36142
100.8 Marker36139
101.6 Marker36138
102.3 Marker36136
106.7 Marker36135
114.8 Marker36132
120.5 Marker36124
124.5 HAU2021
127.6 Marker36118
127.9 Marker36119
129.0 Marker36117
129.4 Marker36116
129.8 Marker36115
130.9 Marker36114
131.6 Marker36113
133.9 Marker36109
134.7 Marker36112
135.8 Marker36072
154.0 DPL0504
155.6 HAU1058
qPH-D10-1(G)
qNFFB-D10-3(G)
D11.1
44.6 Marker36814
45.3 Marker36813
45.6 Marker36812
46.4 Marker36810
49.1 Marker36809
50.2 Marker36807
57.5 Marker36760
57.8 Marker36759
59.3 Marker36755
62.1 HAU2937
65.2 Marker36737
65.6 Marker36724
67.1 Marker36723
68.6 Marker36722
70.4 Marker36719
78.5 Marker36717
80.3 Marker36711
81.3 Marker36700
81.6 Marker36694
82.3 Marker36693
83.0 Marker36692
83.4 Marker36690
qNFFB-D11-1(G)
qHNFFB-D11-1(G)
D11.2
96.5 Marker36680
97.2 Marker36679
97.9 Marker36678
101.3 Marker36676
102.0 Marker36675
103.0 Marker36674
103.7 Marker36673
104.7 Marker36672
105.4 Marker36670
106.4 Marker36669
107.5 Marker36668
108.3 Marker36667
109.0 Marker36666
111.3 Marker36665
115.7 Marker36663
119.2 Marker36662
120.3 Marker36661
122.6 Marker36659
123.7 Marker36657
124.1 Marker36656
126.8 Marker36655
131.4 Marker36645
qHNFFB-D11-2(G)
qNFFB-D11-2(G)
D12.1
31.0 Marker37381
31.9 Marker37437
33.9 Marker37448
35.4 Marker37449
40.7 Marker37450
42.2 Marker37452
43.3 Marker37460
45.2 Marker37461
47.8 Marker37465
49.0 Marker37480
49.5 Marker37481
50.1 Marker37514
51.0 Marker37488
51.3 Marker37496
51.6 Marker37506
51.9 Marker37509
52.2 Marker37487
52.9 Marker37534
53.5 Marker37537
54.3 Marker37538
54.6 Marker37545
55.1 Marker37546
55.5 Marker37548
55.9 Marker37550
56.2 Marker37560
56.4 Marker37561
56.5 Marker37563
59.3 Marker37573
62.8 Marker37576
63.9 Marker37579
64.7 Marker37580
65.0 Marker37581
65.4 Marker37593
65.8 Marker37589
66.5 Marker37599
66.9 Marker37600
67.2 Marker37601
68.0 Marker37604
70.3 Marker37606
71.8 Marker37609
qFT-D12-1(C)
qPH-D12-1(G)
qPH-D12-2(G)

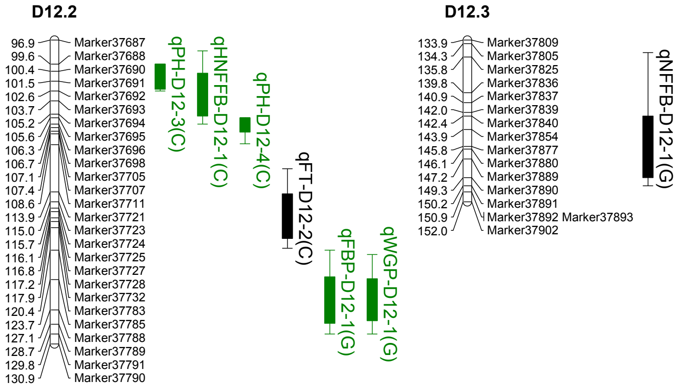

D12.2
96.9 Marker37687
99.6 Marker37688
100.4 Marker37690
101.5 Marker37691
102.6 Marker37692
103.7 Marker37693
105.2 Marker37694
105.6 Marker37695
106.3 Marker37696
106.7 Marker37698
107.1 Marker37705
107.4 Marker37707
108.6 Marker37711
113.9 Marker37721
115.0 Marker37723
115.7 Marker37724
116.1 Marker37725
116.8 Marker37727
117.2 Marker37728
117.9 Marker37732
120.4 Marker37783
123.7 Marker37785
127.1 Marker37788
128.7 Marker37789
129.8 Marker37791
130.9 Marker37790
qPH-D12-3(C)
qHNFFB-D12-1(C)
qPH-D12-4(C)
qFT-D12-2(C)
qFBP-D12-1(G)
qWGP-D12-1(G)
D12.3
133.9 Marker37809
134.3 Marker37805
135.8 Marker37825
139.8 Marker37836
140.9 Marker37837
142.0 Marker37839
142.4 Marker37840
143.9 Marker37854
145.8 Marker37877
146.1 Marker37880
147.2 Marker37889
149.3 Marker37890
150.2 Marker37891
150.9 Marker37892 Marker37893
152.0 Marker37902
qNFFB-D12-1(G)

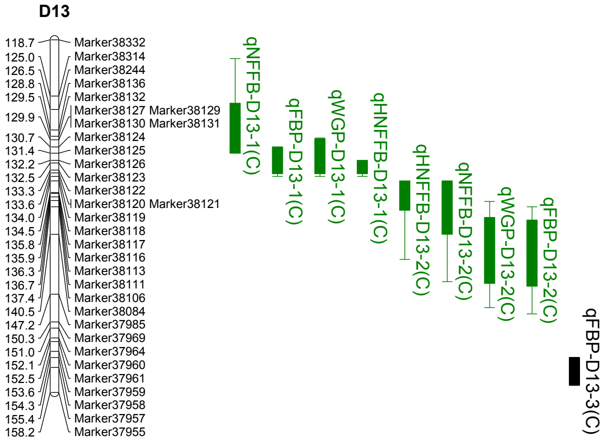

D13
118.7 Marker38332
125.0 Marker38314
126.5 Marker38244
128.8 Marker38136
129.5 Marker38132
129.9 Marker38127 Marker38129
Marker38130 Marker38131
130.7 Marker38124
131.4 Marker38125
132.2 Marker38126
132.5 Marker38123
133.3 Marker38122
133.6 Marker38120 Marker38121
134.0 Marker38119
134.5 Marker38118
135.8 Marker38117
135.9 Marker38116
136.3 Marker38113
136.7 Marker38111
137.4 Marker38106
140.5 Marker38084
147.2 Marker37985
150.3 Marker37969
151.0 Marker37964
152.1 Marker37960
152.5 Marker37961
153.6 Marker37959
154.3 Marker37958
155.4 Marker37957
158.2 Marker37955
qNFFB-D13-1(C)
qFBP-D13-1(C)
qWGP-D13-1(C)
qHNFFB-D13-1(C)
qHNFFB-D13-2(C)
qNFFB-D13-2(C)
qWGP-D13-2(C)
qFBP-D13-2(C)
qFBP-D13-3(C)
