# Supplementary material for: High-density linkage map construction and QTL analysis for earliness-related traits in Gossypium hirsutum L
Source: BMC Genomics. 2016 Nov 11;17:909. doi: 10.1186/s12864-016-3269-y (PMC5106845; doi:10.1186/s12864-016-3269-y)
Supplement: Additional file 9: — Recombinants in the RIL population at locus qFT-D3-3 defining the core region between Marker25957 and Marker25965. CCRI36 is the maternal cultivar; G2005 is the paternal line; b is the homozygous allele conferred by CCRI36; a is the homozygous allele conferred by G2005; and h is heterozygous. (PDF 124 kb) [file 12864_2016_3269_MOESM9_ESM.pdf]

Recombinants in the RIL population at the locus of qFT-D3-3 defined the region between Marker25957 and Marker25965

RIL line	Marker25957	Marker25958	Marker25959	Marker25963	Marker25964	Marker25965	2013FT	Class
174	b	b	b	b	b	b	65.67	early
182	b	b	b	b	b	b	65.67	early
149	a	b	b	b	b	b	65.67	early
192	a	a	b	b	h	b	67	early
76	b	b	b	a	a	a	68	early
CCRI36	b	b	b	b	b	b	65.67	early
G2005	a	a	a	a	a	a	75.33	late
44	a	a	a	b	b	b	70.67	late
138	a	a	a	b	b	b	72.33	late
69	a	a	a	a	a	b	71.33	late
23	b	a	a	a	a	a	70.67	late
52	b	a	h	a	a	a	78.67	late
171	a	a	a	a	a	a	76	late
49	a	a	a	a	a	a	76.33	late
