# Supplementary material for: High-density linkage map construction and QTL analysis for earliness-related traits in Gossypium hirsutum L
Source: BMC Genomics. 2016 Nov 11;17:909. doi: 10.1186/s12864-016-3269-y (PMC5106845; doi:10.1186/s12864-016-3269-y)
Supplement: Additional file 13: — Heat maps of the 26 linkage groups. (PDF 4231 kb) [file 12864_2016_3269_MOESM13_ESM.pdf]

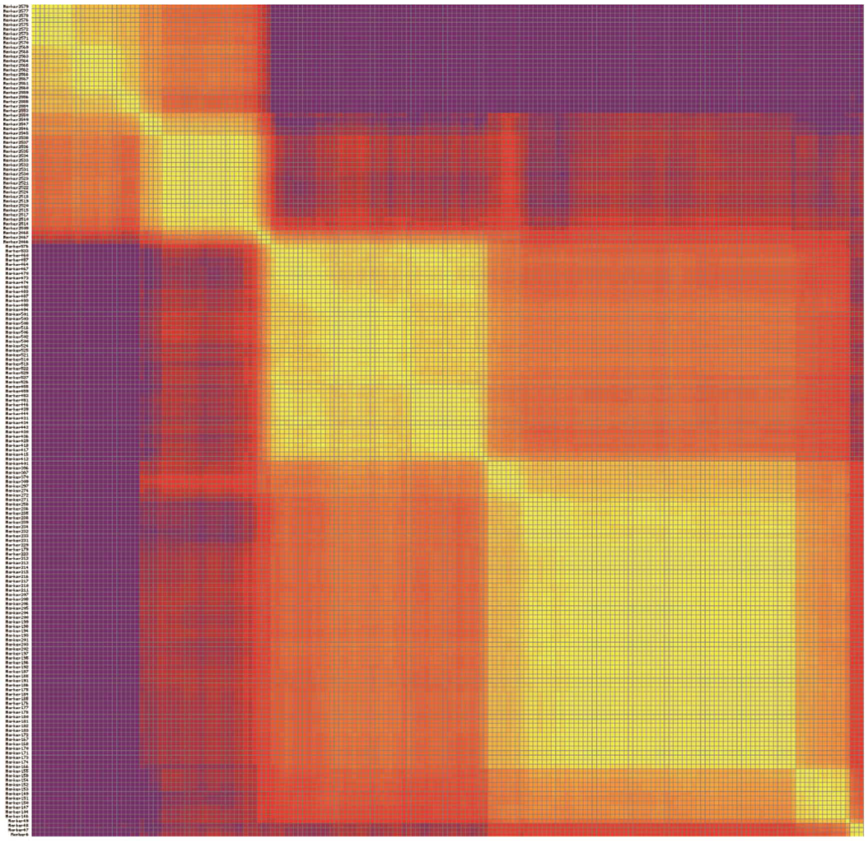

Chromosome A1

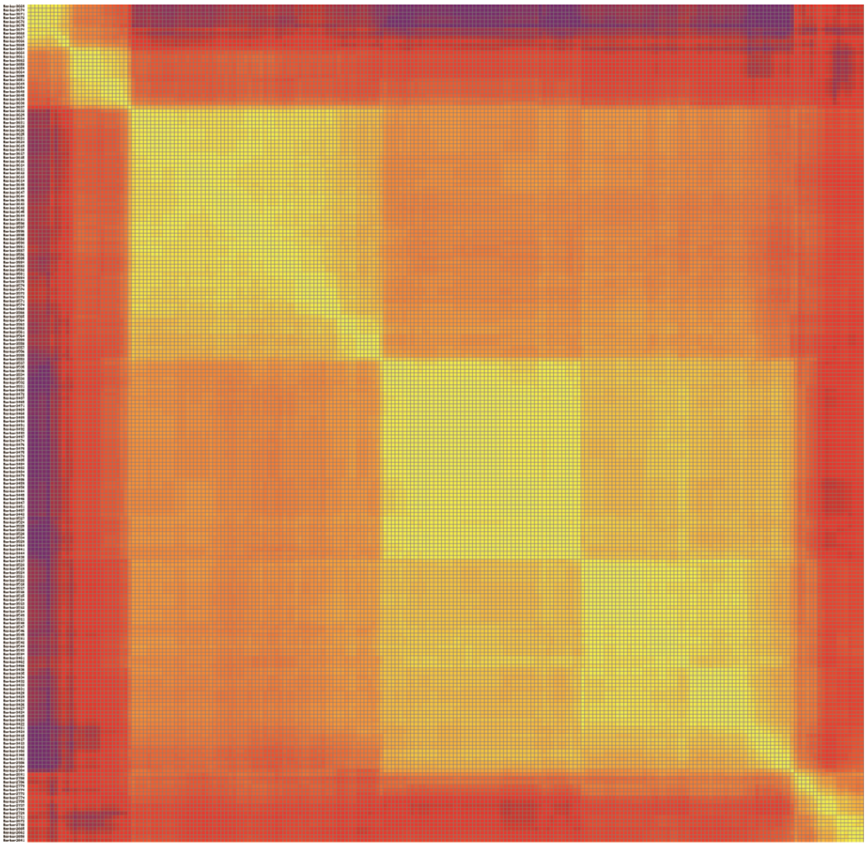

Chromosome A2

Supplemental file 4 Heat maps of the 26 linkage groups. Color from yellow to red and then purple indicates the increase of recombination frequency.

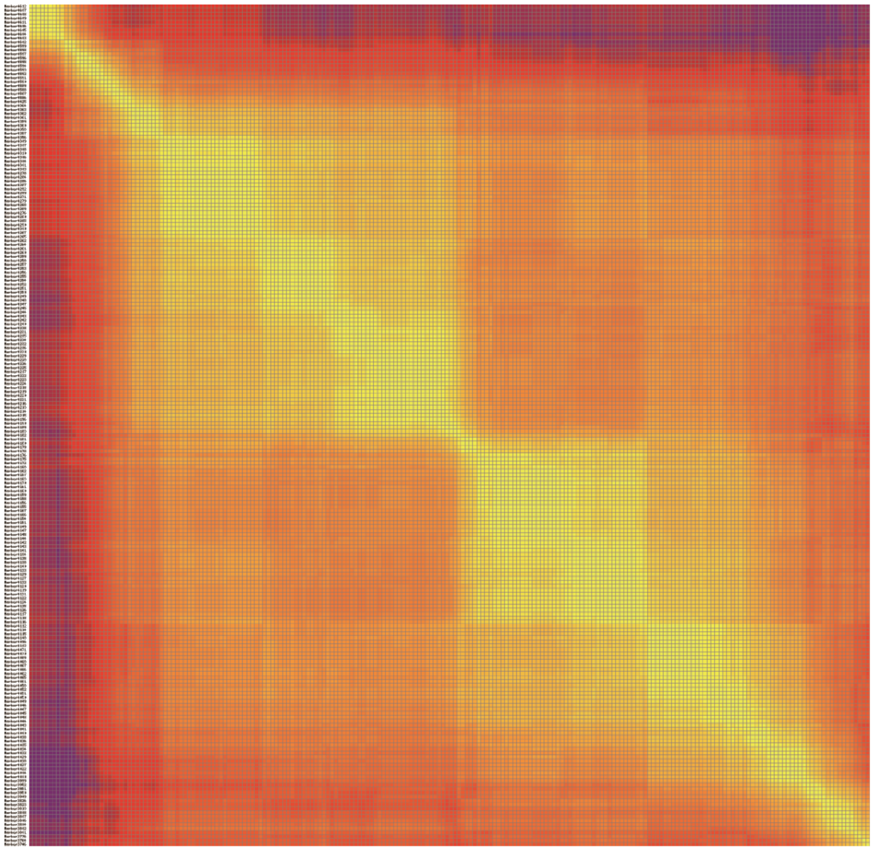

Chromosome A3

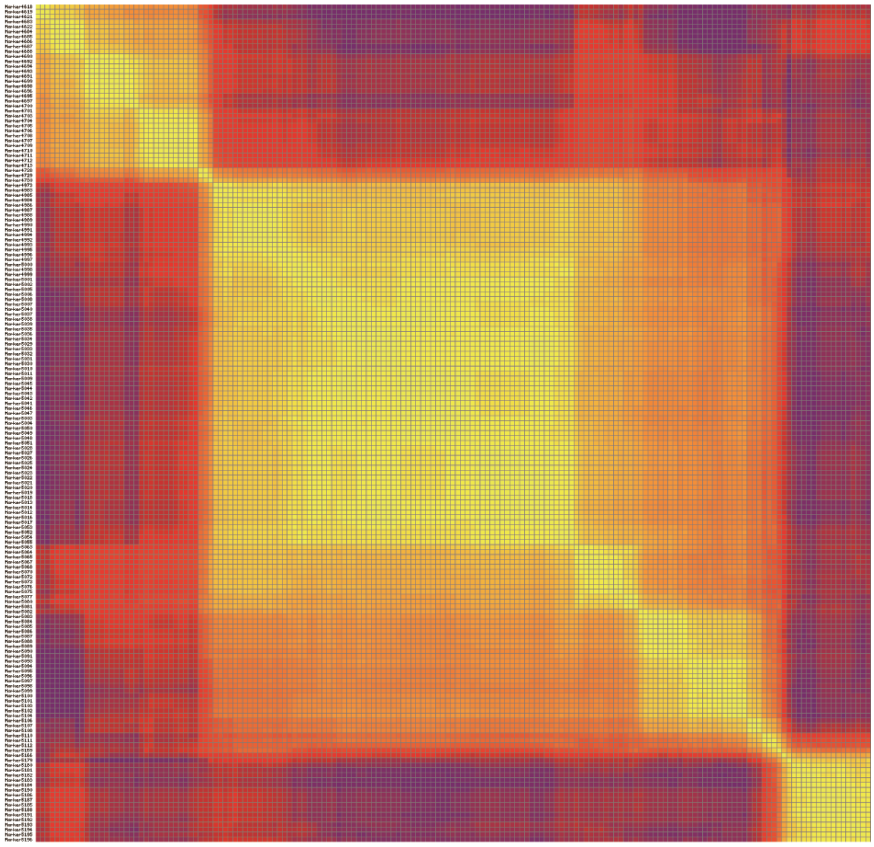

Chromosome A4

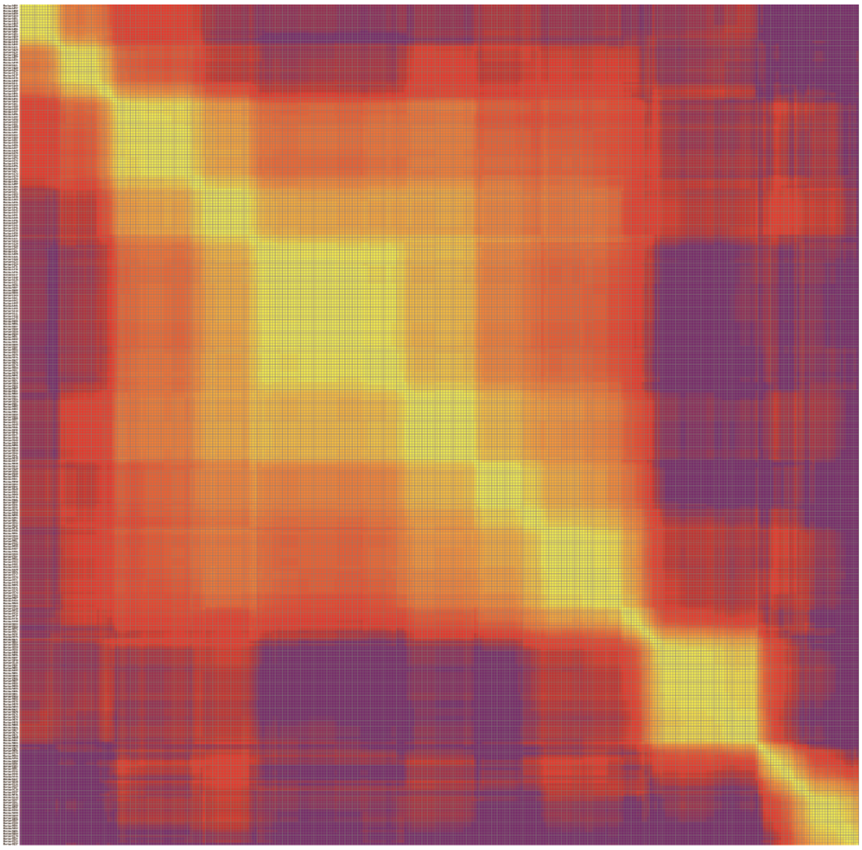

Chromosome A5

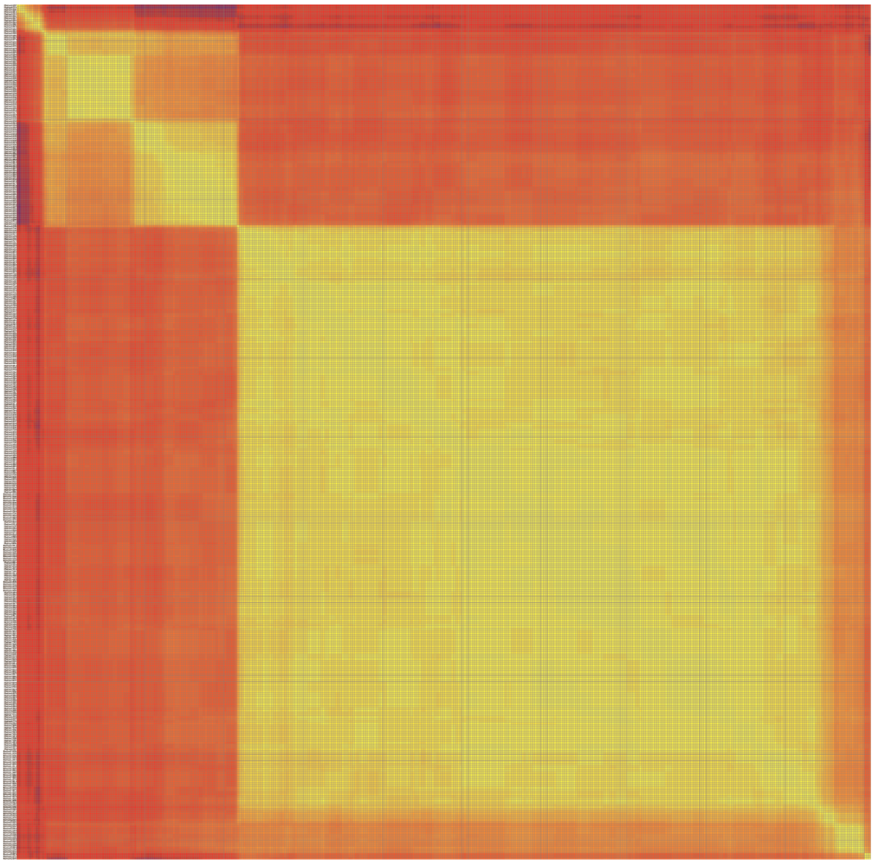

Chromosome A6

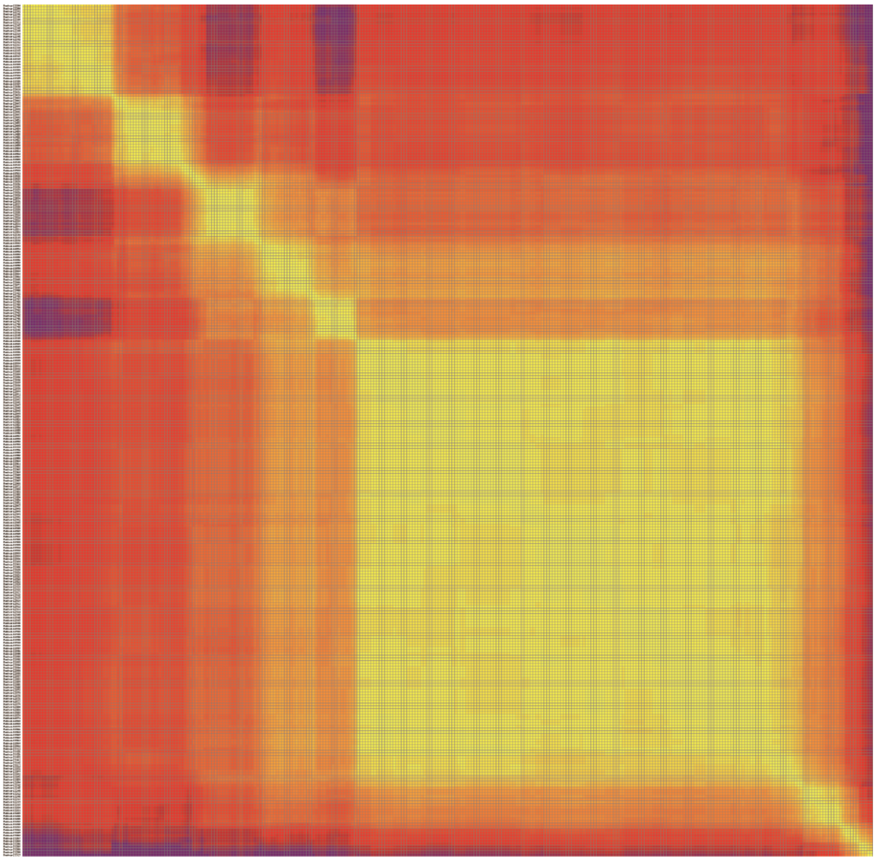

Chromosome A7

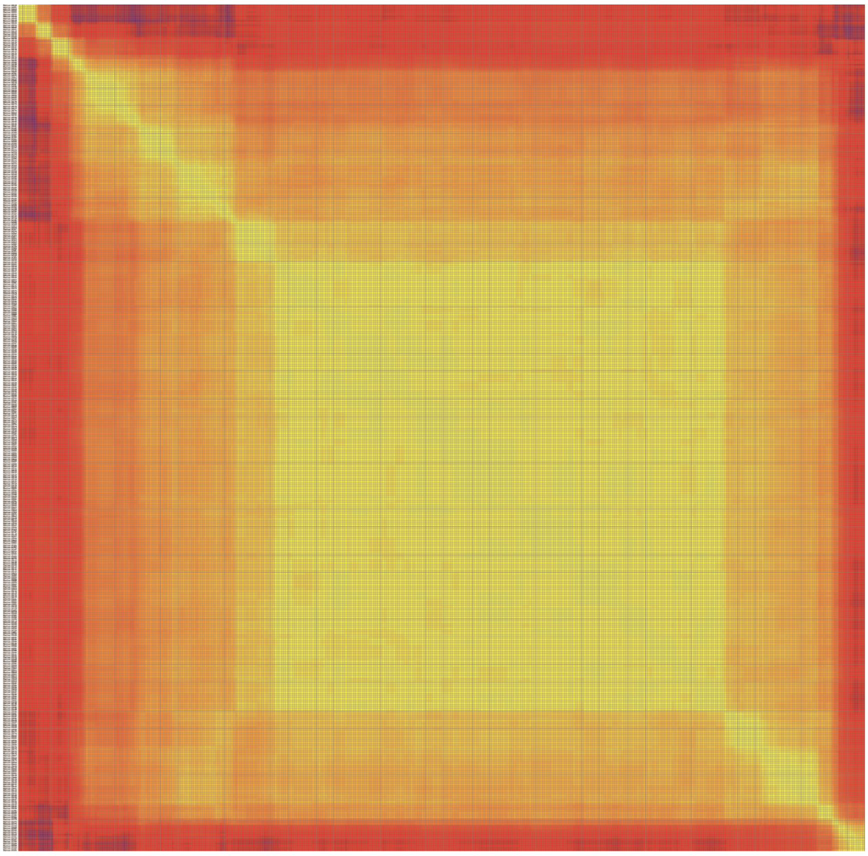

Chromosome A8

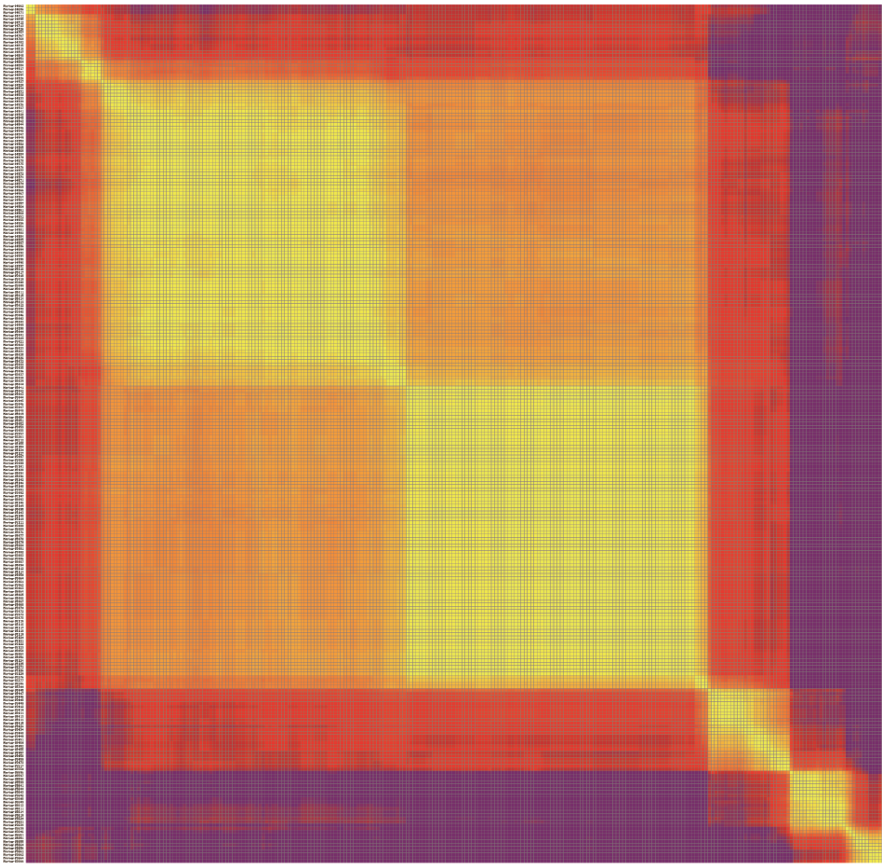

Chromosome A9

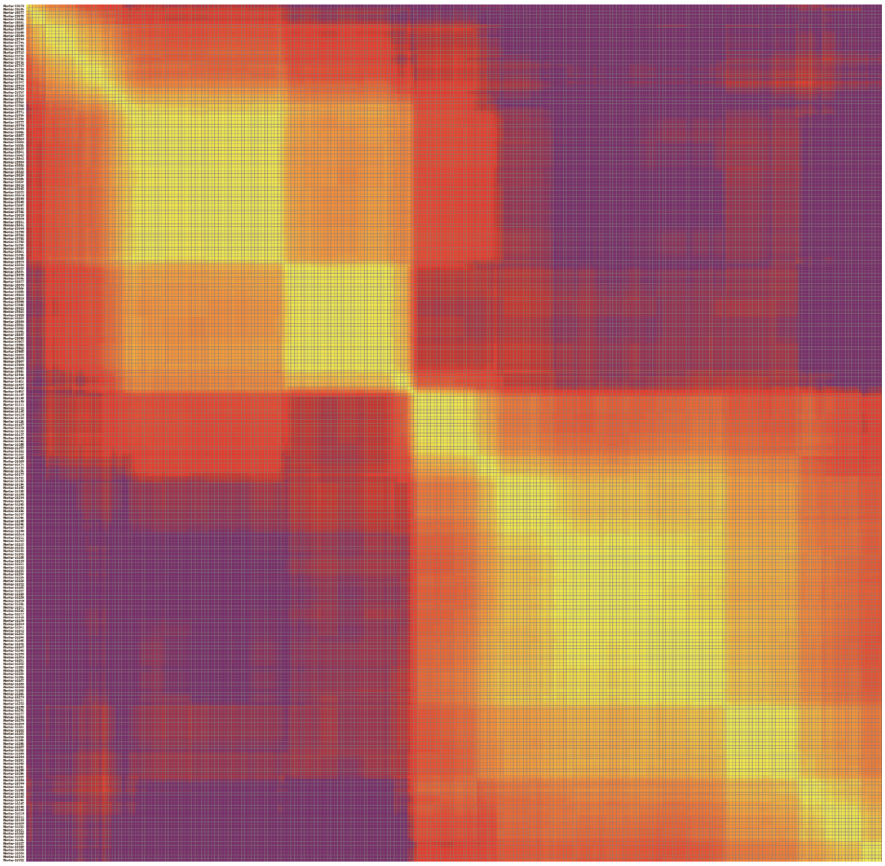

Chromosome A10

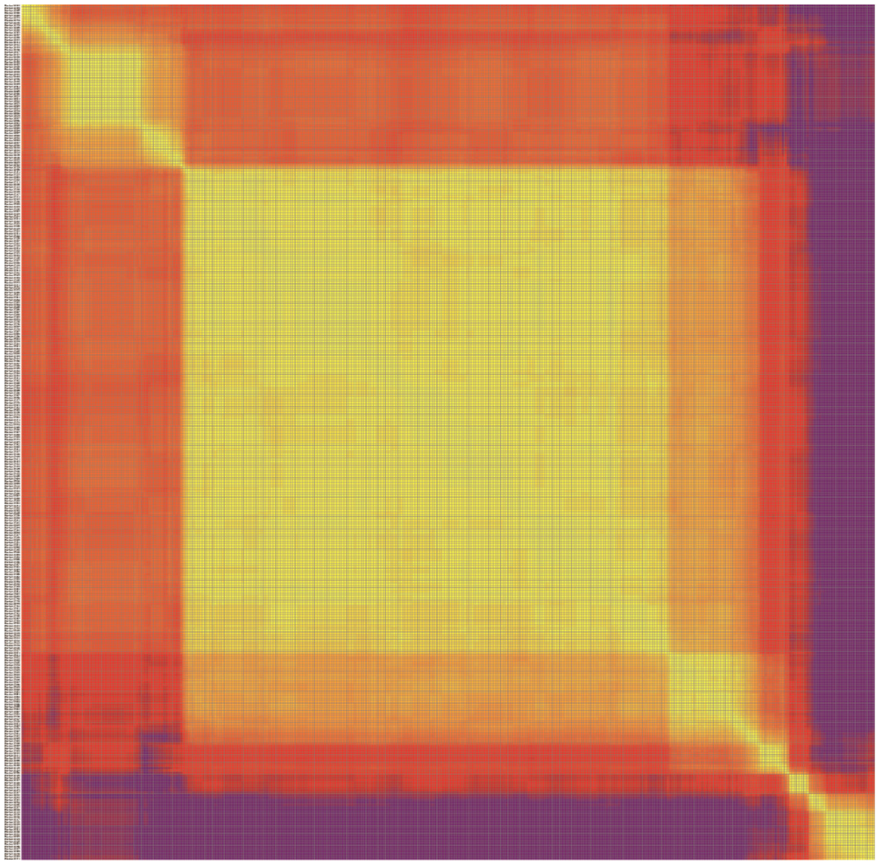

Chromosome A11

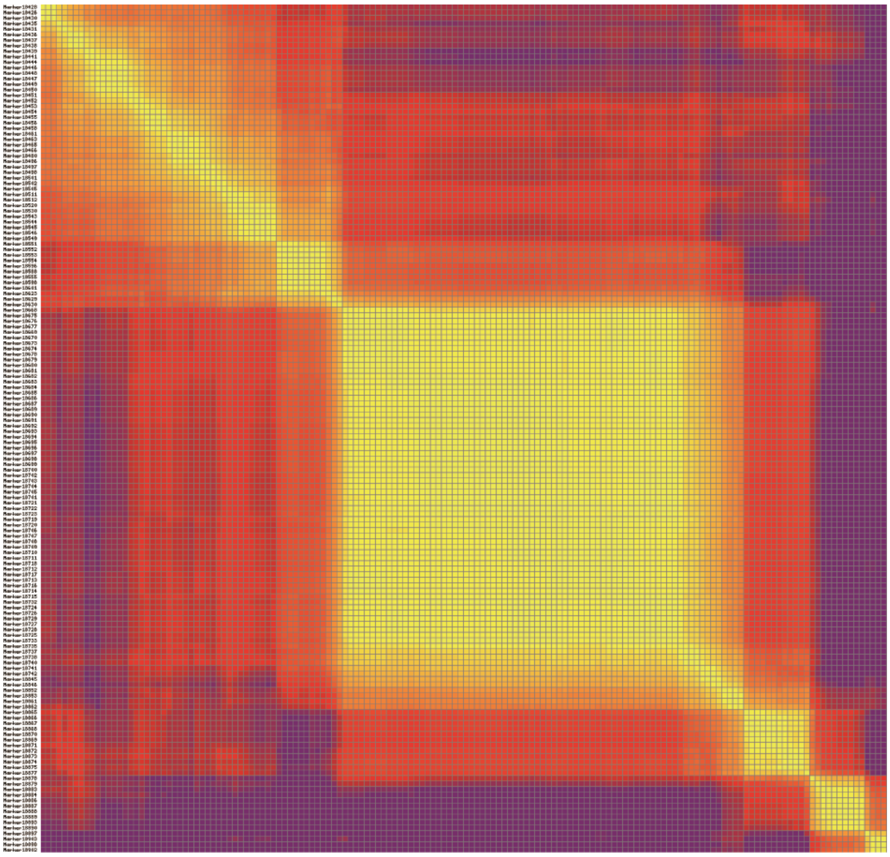

Chromosome A12

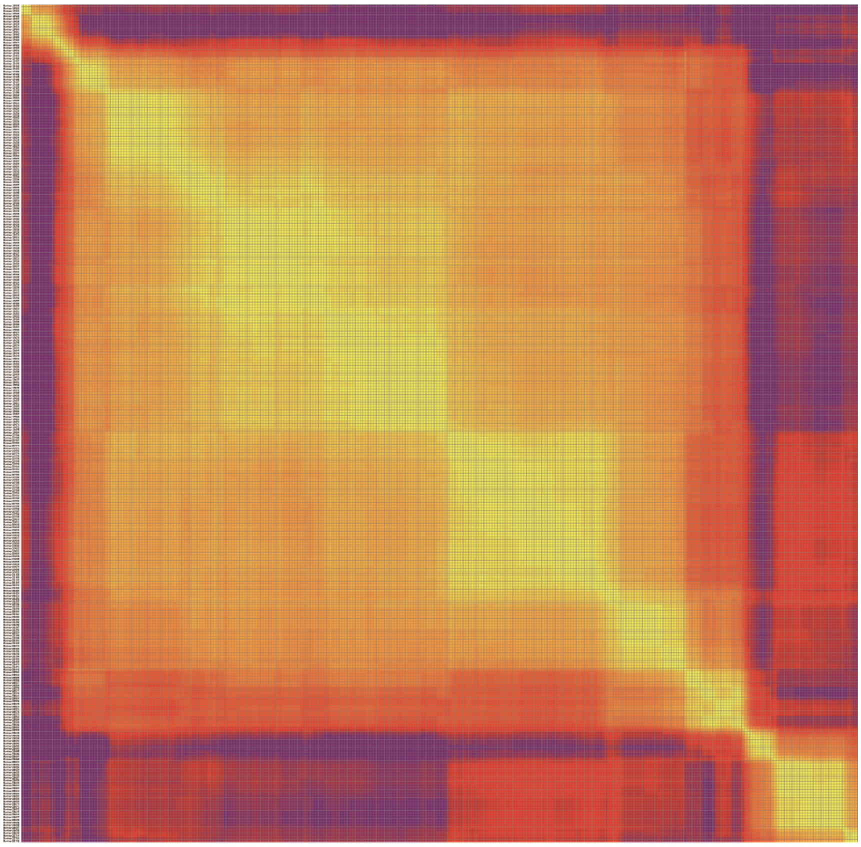

Chromosome A13

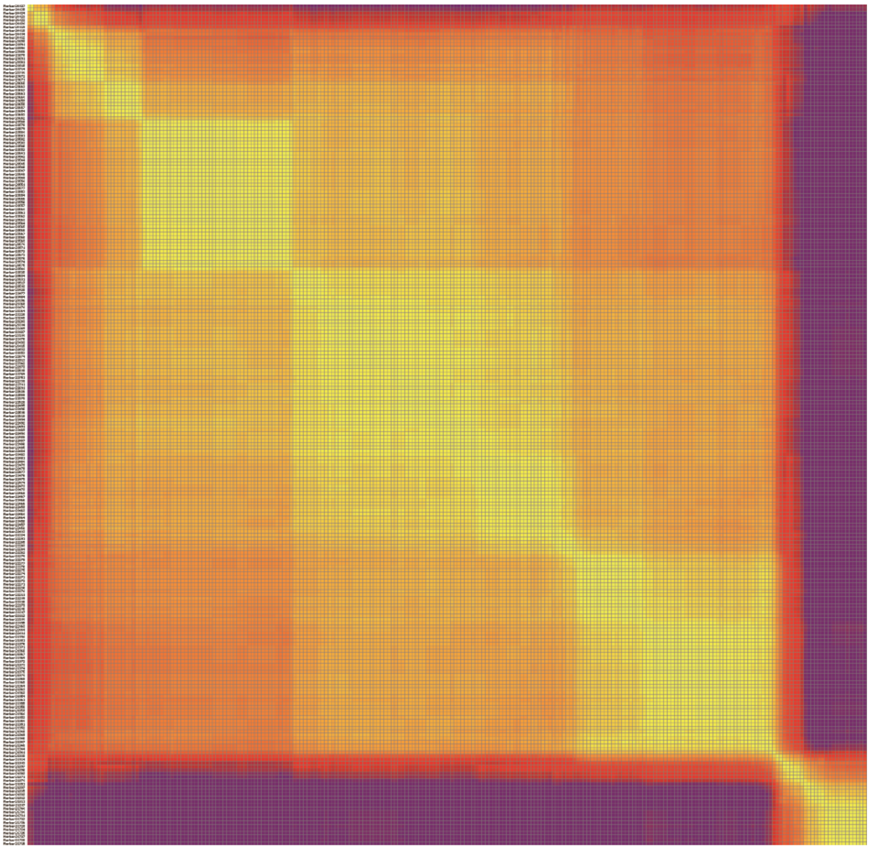

Chromosome D1

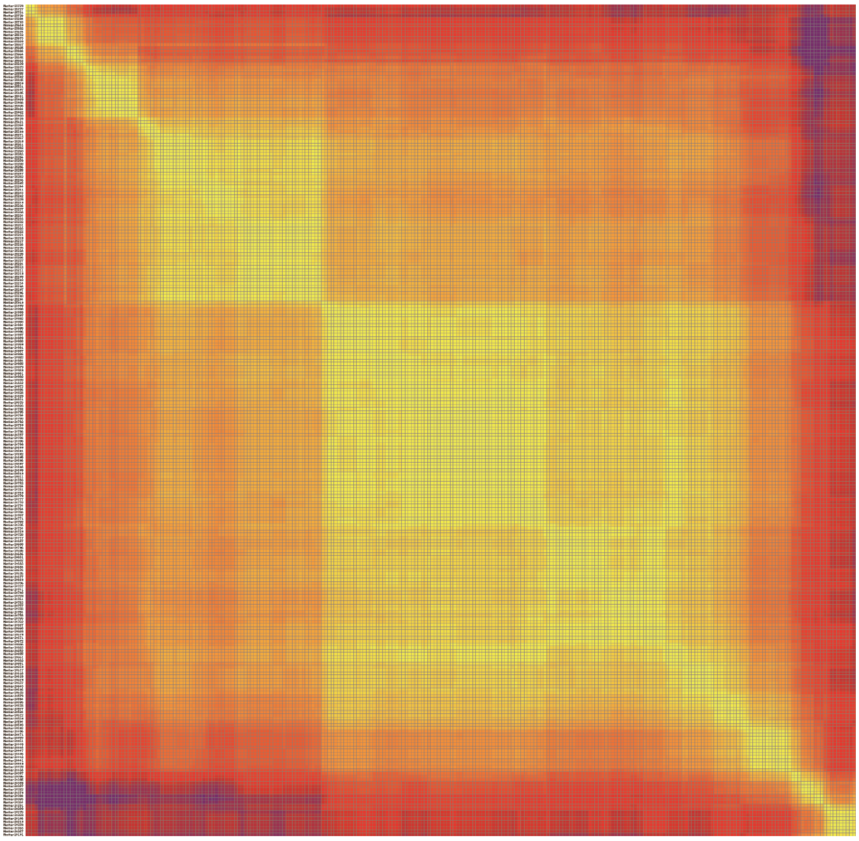

Chromosome D2

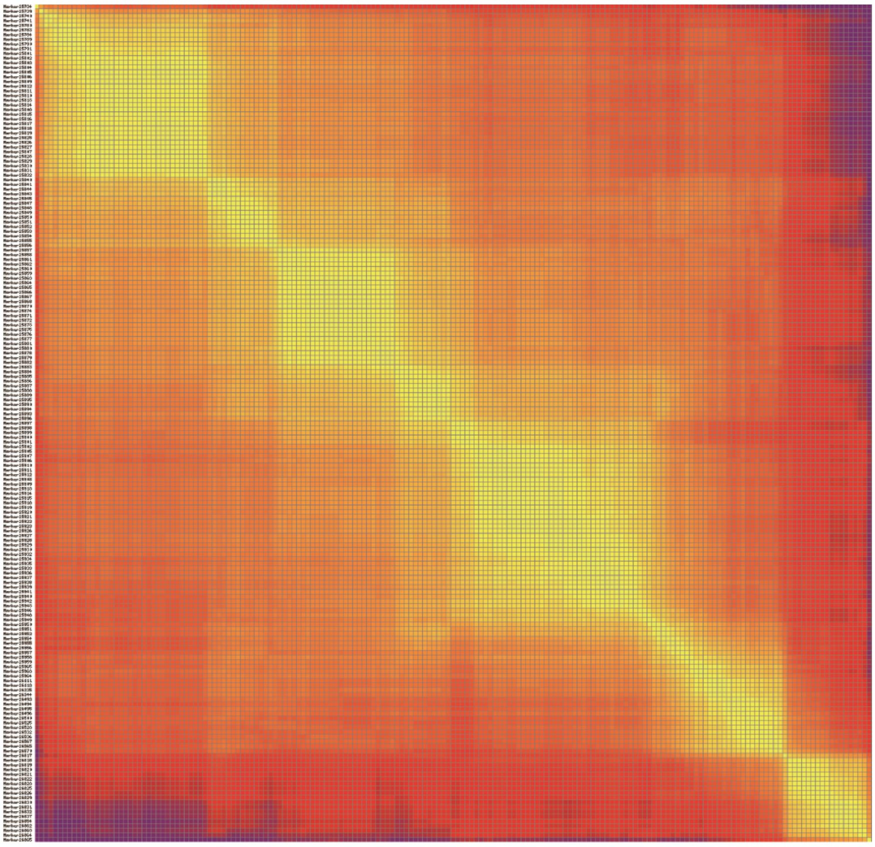

Chromosome D3

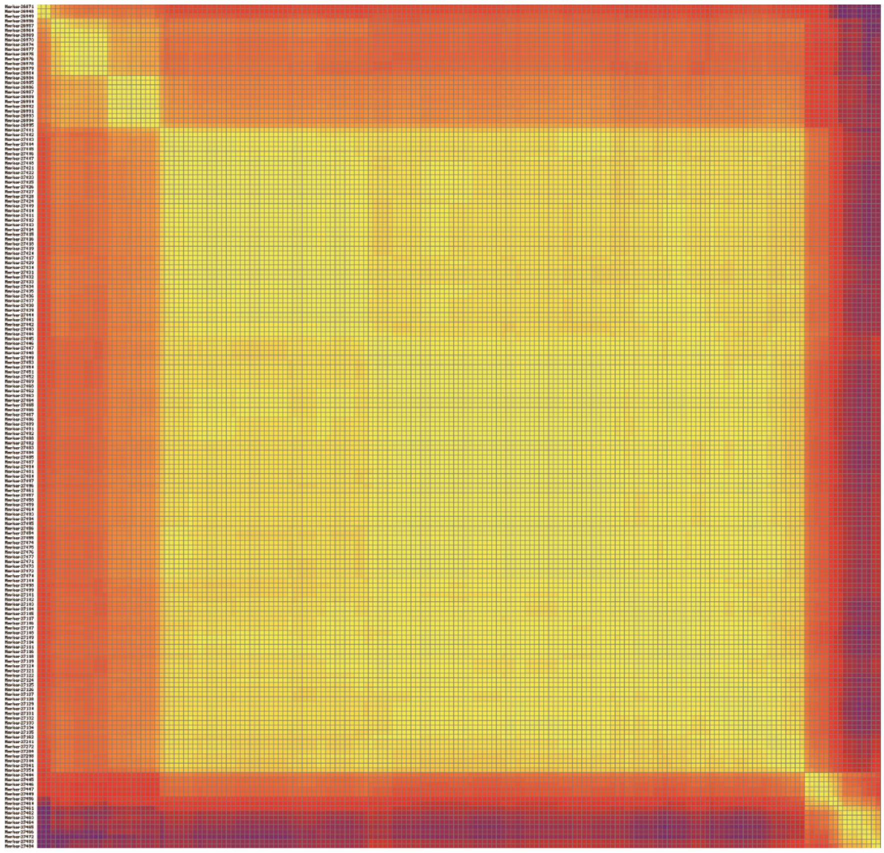

Chromosome D4

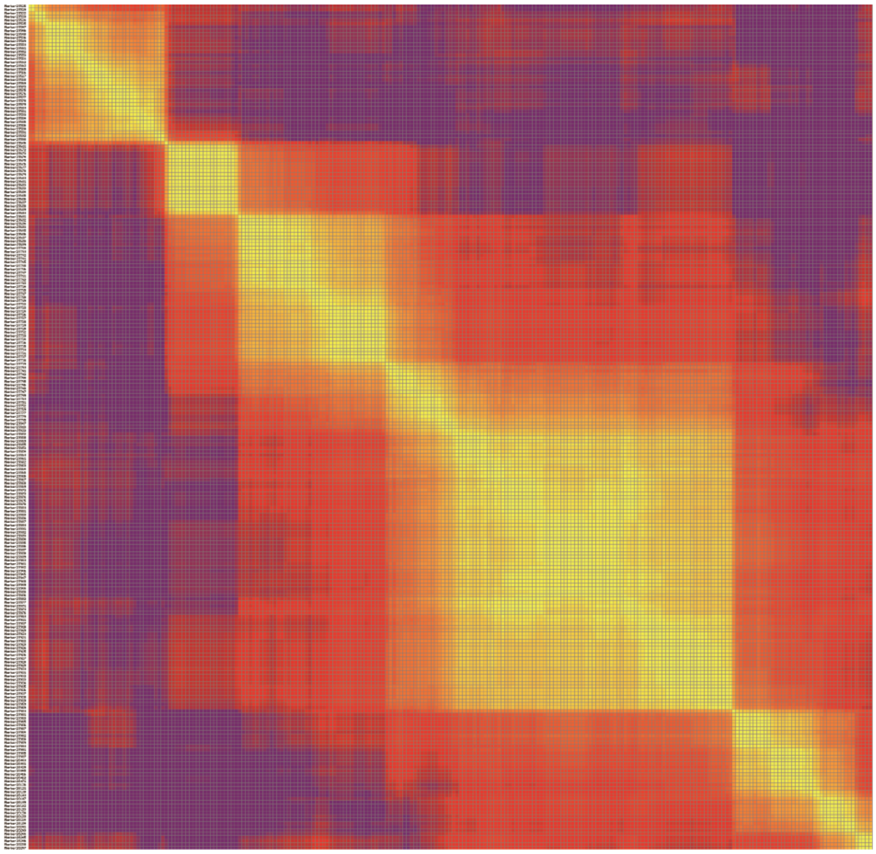

Chromosome D5

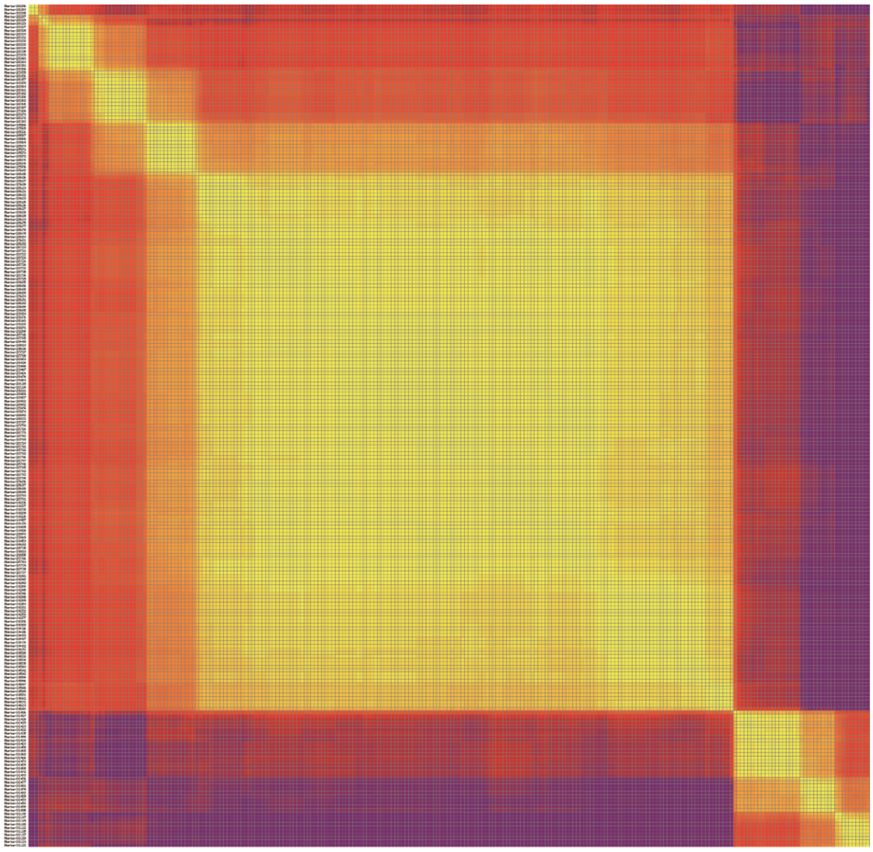

Chromosome D6

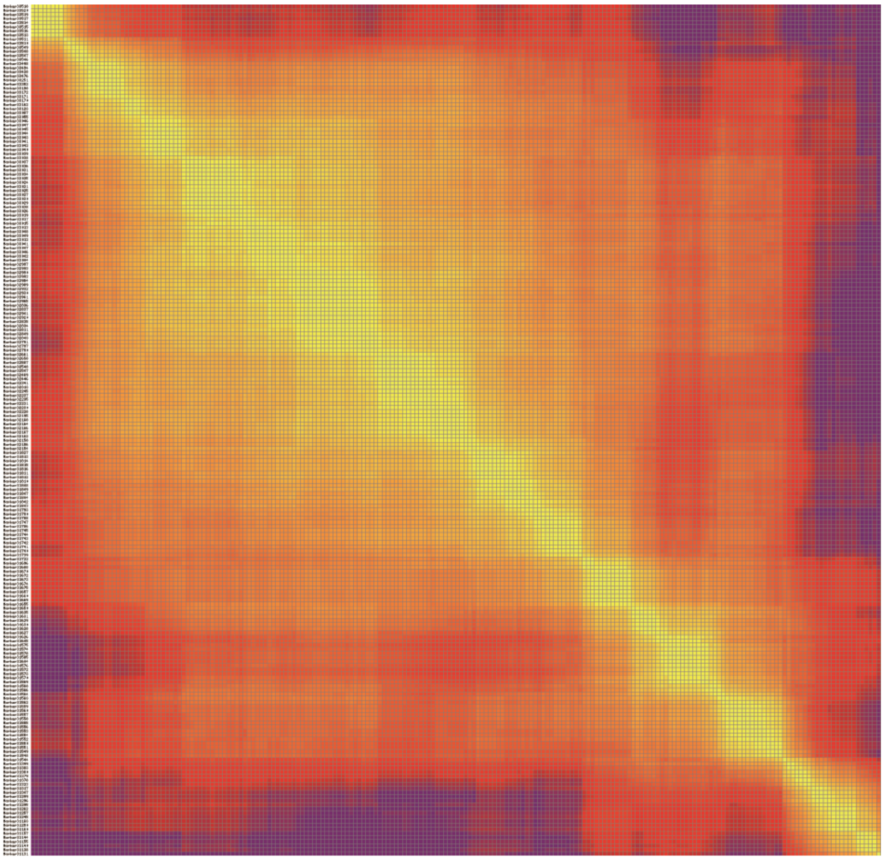

Chromosome D7

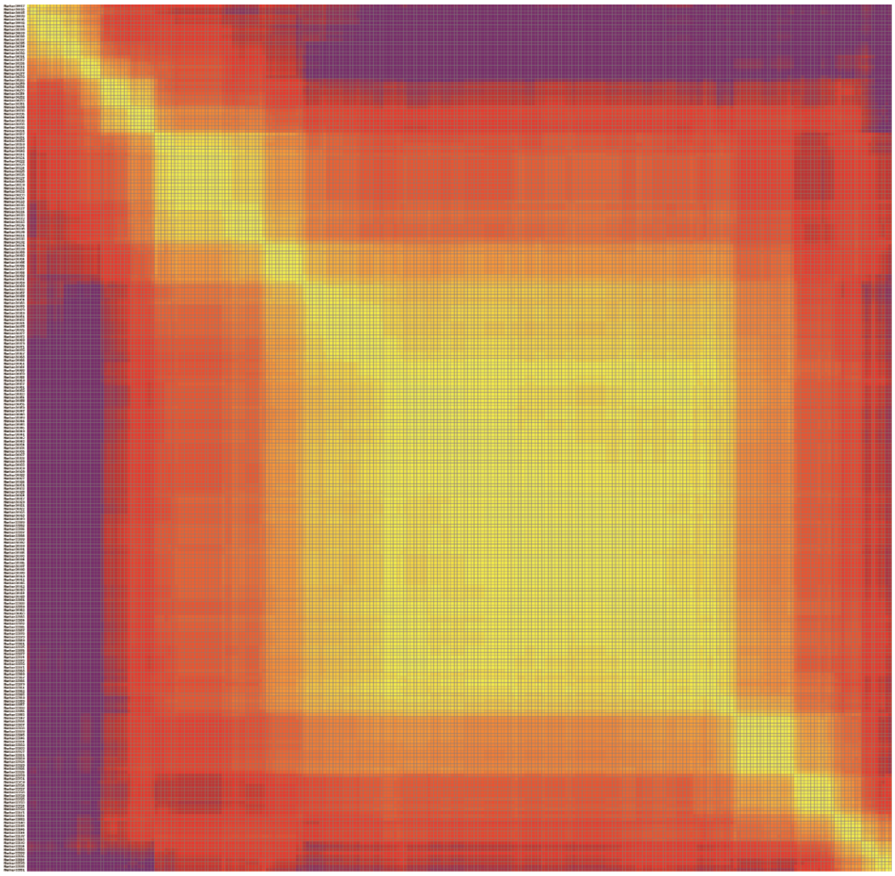

Chromosome D8

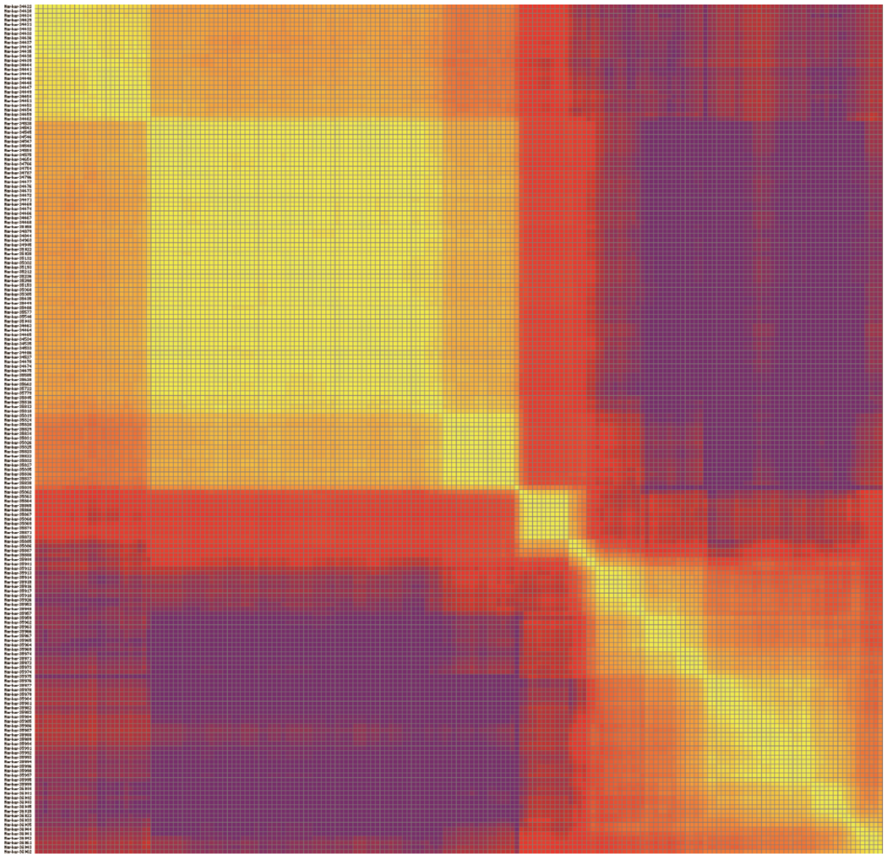

Chromosome D9

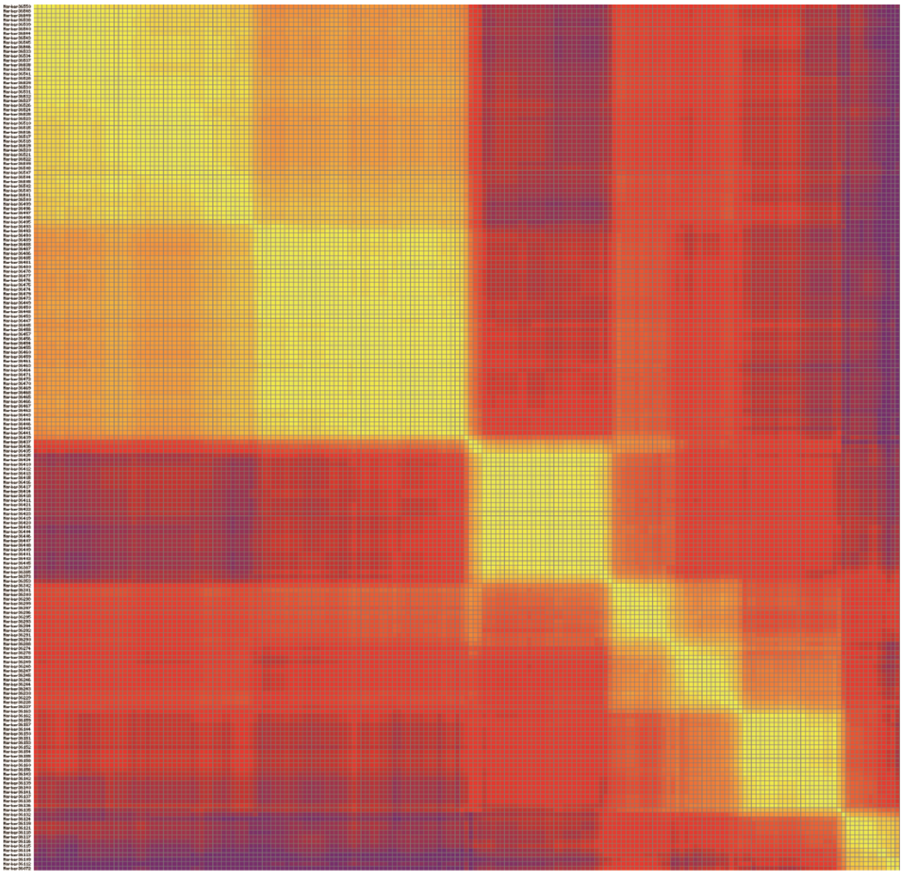

Chromosome D10

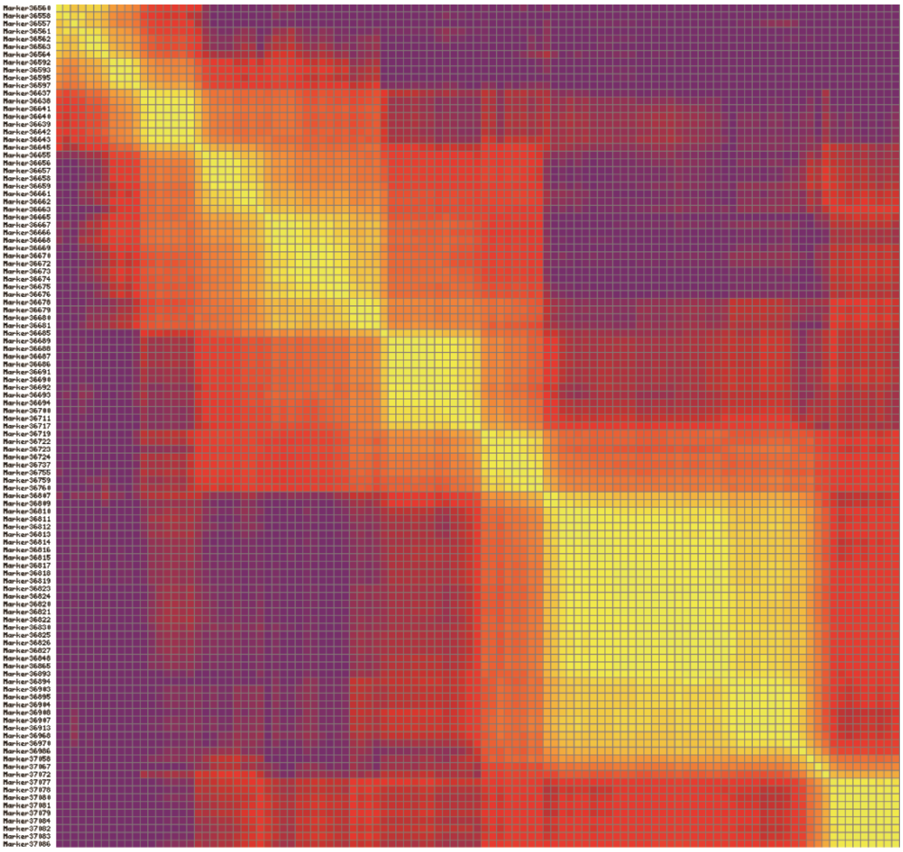

Chromosome D11

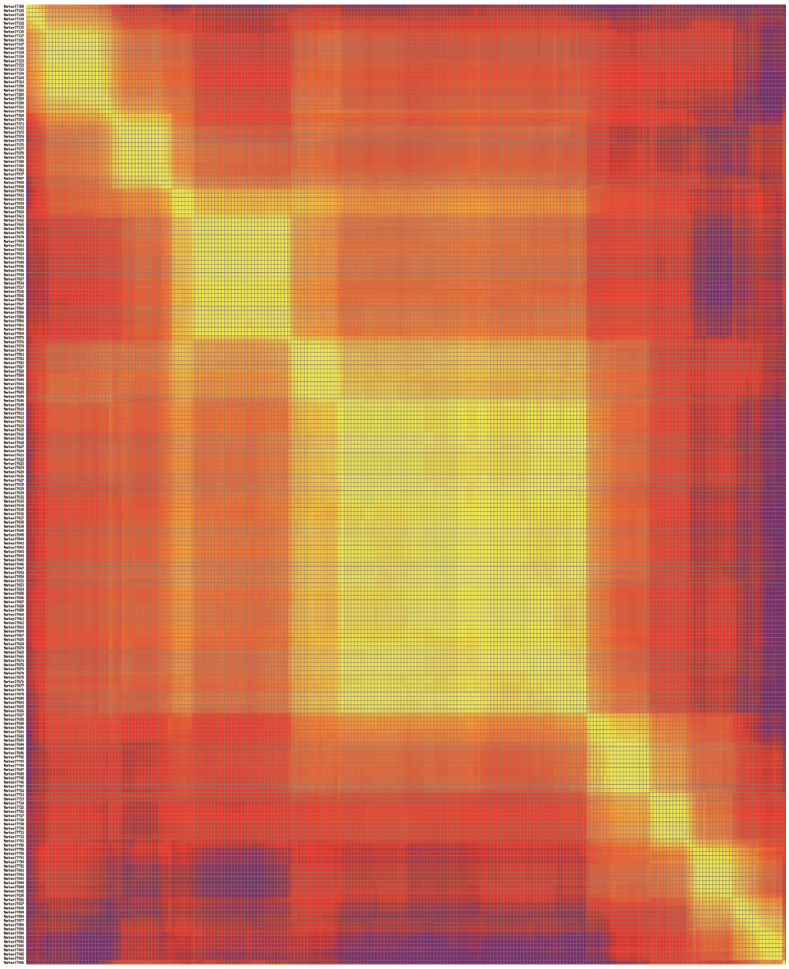

Chromosome D12

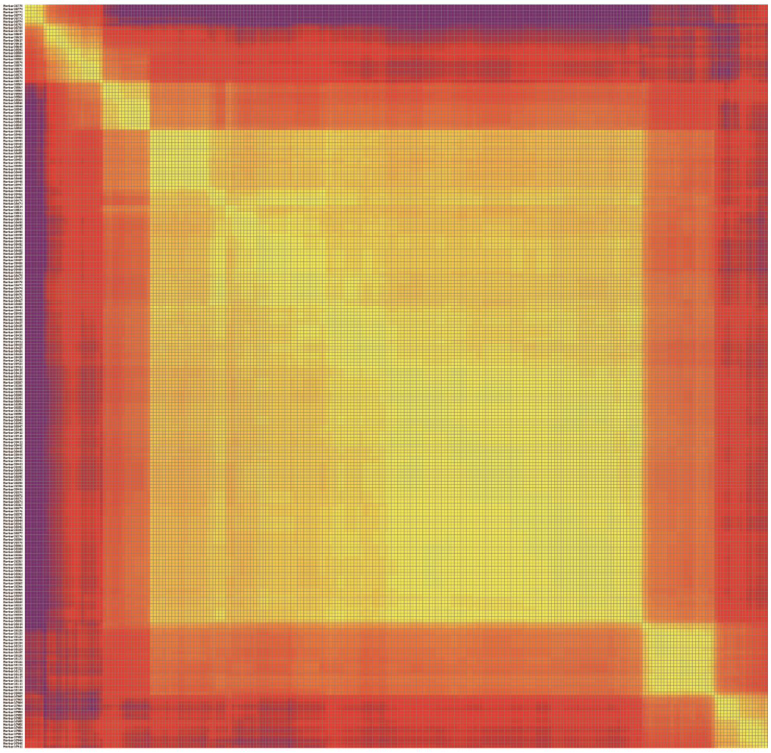

Chromosome D13
